# Effect modifiers of the temperature-mortality association for general and older adults population of Brazil’s metropolitan areas

**DOI:** 10.1590/0102-311XEN042524

**Published:** 2025-02-24

**Authors:** Cristiane Aschidamini, Antônio Carlos Monteiro Ponce de Leon

**Affiliations:** 1 Universidade do Estado do Amazonas, Manaus, Brasil.; 2 Centro Biomédico, Universidade do Estado do Rio de Janeiro, Rio de Janeiro, Brasil.

**Keywords:** Temperatures, Mortality, Epidemiologic Effect Modifier, Climate Effects, Temperatura Ambiental, Mortalidad, Modificador del Efecto Epidemiológico, Efectos del Clima

## Abstract

Ambient temperature effect on mortality varies between places and populations, suggesting the existence of effect modifiers for this association. This study analyzes the influence of geographic, urban, and socioeconomic factors on the ambient temperature effect on non-accidental mortality in the general and older adults population of Brazilian metropolitan areas, and on that associated with circulatory, respiratory, and other mortality in older adults. Effects of this association were estimated for each group in 42 locations using a generalized additive model combined with the nonlinear distributed lag model. A meta-analysis was then performed to estimate the effects at the national and regional levels. Meta-regression determined the influence of effect modifiers. Estimated relative risks of the temperature-mortality association varied between locations in the Brazilian territory. Heat effects on non-accidental mortality at the national level were 1.09 (95%CI: 1.04-1.15) and 1.13 (95%CI: 1.07-1.20) for the General and Older Adult groups, respectively. Cold effects were 1.26 (95%CI: 1.21-1.32) and 1.30 (95%CI: 1.24-1.36) for the General and Older Adult groups, respectively. We observed a greater effect of cold than heat in both groups. For all causes of death, effects of heat and cold were greater in the Southeast and South Brazil. Amplitude of the mean temperature was the factor that best explained the heterogeneity between locations, followed by latitude, income and schooling. Hence, implementing adaptive measures to reduce the ambient temperature effects on mortality depends on the profile of each location.

## Abstract (es)

El efecto de la temperatura ambiente sobre la mortalidad varía entre sitios y poblaciones, lo que sugiere la presencia de modificadores del efecto de esta asociación. El objetivo de este estudio fue analizar la influencia de los factores geográficos, urbanos y socioeconómicos en el efecto de la temperatura ambiente sobre la mortalidad no accidental en la población general y anciana de las regiones metropolitanas brasileñas, y la influencia asociada con las causas de mortalidad circulatoria, respiratoria u otra en los ancianos. Los efectos de esta asociación se estimaron para cada grupo en 42 sitios mediante un modelo aditivo generalizado combinado con el modelo no lineal distribuido. A continuación, se realizó un metaanálisis para estimar los efectos a nivel Brasil y sus regiones. La influencia de los modificadores del efecto se determinó mediante metarregresión. Los riesgos relativos estimados de la asociación temperatura-mortalidad variaron entre las ubicaciones en el territorio brasileño. Los efectos del calor sobre la mortalidad no accidental a nivel nacional fueron de 1,09 (IC95%: 1,04-1,15) y de 1,13 (IC95%: 1,07-1,20) para el grupo General y Ancianos, respectivamente. Los efectos del frío fueron 1,26 (IC95%: 1,21-1,32) y 1,30 (IC95%: 1,24-1,36) para el grupo General y Anciano, respectivamente. Hay un mayor efecto del frío que del calor en ambos grupos. Para todas las causas de muerte, los efectos del calor y del frío fueron mayores en las regiones Sudeste y Sur de Brasil. El factor que explicó mejor la heterogeneidad entre los locales fue el rango de temperatura media, seguido de la latitud, los ingresos y el nivel de estudios. Por lo tanto, la implementación de medidas de adaptación para reducir los efectos de la temperatura ambiente sobre la mortalidad depende del perfil de cada lugar.

## Introduction

High and low ambient temperatures are related to increases in medical emergencies, hospitalization, and deaths ^1^. Temperature-mortality association was observed for overall mortality ^1^ ^,^ ^2^, cardiovascular disease ^3^ ^,^ ^4^, respiratory diseases ^5^, and cerebrovascular diseases ^4^ ^,^ ^6^, in locations across Europe ^7^, Asia ^8^ ^,^ ^9^, Africa ^10^, North America ^11^ and Latin America ^12^, thus highlighting a global health issue that can be amplified by climate change events ^13^.

Variations of these mortality risks related to ambient temperature between locations ^4^ ^,^ ^9^ ^,^ ^11^ ^,^ ^12^ ^,^ ^14^ ^,^ ^15^ ^,^ ^16^ may be due to the variability of individual and community characteristics ^17^, socioeconomic characteristics ^7^, geographic aspects ^16^ or different adaptation responses ^2^. These effect modifiers of the temperature-mortality association were investigated in previous studies, especially in areas from North America, Asia, and Europe ^17^. However, these studies showed divergent results and few compared different geographic and climatic regions ^17^ ^,^ ^18^.

Brazil is also vulnerable to the effects of extreme temperatures, with temperature-related mortality ranging from 2.04% to 7.08% between Brazilian capitals ^2^. Such heat stress conditions are associated with higher hospitalization rates ^19^ ^,^ ^20^ and mortality due to cardiovascular and respiratory diseases ^21^.

Located in South America, Brazil has over 203 million inhabitants distributed across more than 8.5 million km^2^. As an emerging country, few locations in its territory have consistent active policies for climate change adaptation or mitigation ^22^. With a large territorial extension, socio-environmental and sociodemographic diversity, Brazil is an appropriate place of study to expand research in this area.

Few national studies address the effect of extreme temperatures on mortality ^23^, especially on Brazilian older adults, an expanding group that is vulnerable to ambient temperature effects ^17^ ^,^ ^23^. Additionally, these studies fail to address temperature effects on populations living in metropolitan areas. This area of great urbanization encompasses a set of contiguous municipalities socioeconomically integrated into a central city that share public services and infrastructure and concentrate a third of Brazil’s population, 60% of the national gross domestic product (GDP) and 70% of urban poverty ^24^. Studies in these areas enable analyzing data from a larger contingent of urban dwellers, a factor also related to greater vulnerability to extreme temperatures ^18^.

Understanding this association and its modifying factors enables planning appropriate interventions for subpopulations with different vulnerability statuses and providing reliable predictions of climate change effects ^2^ ^,^ ^17^.

Seeking to contribute to this issue, the present study estimated the effect of ambient temperature on non-accidental mortality among the general population and older adults across the Brazilian territory, and analyzed the influence of geographic, urban and socioeconomic diversity on this association.

## Methods

### Study design

A time series analysis of daily mortality and meteorological data was performed from January 1, 2000, to December 31, 2014, for 45 Brazilian metropolitan areas located in the Central-West (2), Northeast (16), North (7), Southeast (8) and South (12). Other 29 metropolitan areas were excluded for lacking a climate data collection station.

### Mortality data

Mortality data were obtained from the Brazilian Mortality Information System (SIM, acronym in Portuguese), Brazilian Health Informatics Department (DATASUS, acronym in Portuguese). Non-accidental daily mortality (General group) is represented by the total count of deaths excluding external causes (International Classification of Diseases, 10th revision of the [ICD-10]: A00-R99). The Older Adult group used data on the deaths of individuals aged 60 years or older.

To verify the diversity of effects between causes of death, we analyzed the association between temperature mortality from circulatory, respiratory and other causes in the Older Adult group. Mortality data were stratified by cause of death, forming the subgroups: Circulatory (diseases of the circulatory system - ICD-10: I00-I99); Respiratory (diseases of the respiratory system - ICD-10: J00-J99), and Other Causes (ICD-10: A00-H95 and K00-R99).

Low number of deaths in the time series may lead to imprecision in the estimates ^25^. Presence of inaccurate data in the first statistical analyses can generate bias in the data estimated in the second phase ^26^. To minimize possible errors in the subsequent combined estimates, exclusion criteria were applied. First, we excluded metropolitan areas with a number of deaths per day lower than 1.5, remaining 43 metropolitan areas for the analyses of the General and Older Adult groups. Finally, one metropolitan area was excluded due to numerical inconsistencies (numerical problems in estimation). For the Older Adult subgroups, with lower death counts, metropolitan areas with a daily mean of deaths below 1 in all 3 subgroups were also excluded, thus totaling 38 metropolitan areas for the subgroup analysis.

### Meteorological data

Mean daily temperature (ºC) was chosen as the exposure variable to be analyzed, as it represents exposure throughout the day and night, having the best performance in predicting temperature effects on mortality ^9^, and because different temperature measurements have similar predictive abilities ^10^ ^,^ ^27^.

Average of the current and previous day was used as the average relative humidity indicator (%). It was included in the analysis as a confounding factor, as in previous studies ^23^ ^,^ ^28^. Humidity influences temperature by modulating thermal sensation ^29^ ^,^ ^30^. It also influences the development of respiratory ^30^ and cardiovascular diseases by affecting heat stress, dehydration and proliferation of disease vectors ^29^ ^,^ ^30^.

Missing data on mean compensated temperature and mean relative humidity could be minimized for five locations that had two or more metropolitan stations. In these, imputation of missing values was performed using the *mtsdi* package ^31^ of the R platform (http://www.r-project.org). A model with nonparametric cubic spline with 8 degrees of freedom (df) predicted the data to be imputed. Imputation occurred only for days in which there was at least one observation per weather station and there could be no loss of data for more than three days.

Previous studies have reported the influence of daily and yearly temperature variations ^28^ ^,^ ^32^, locations, latitude ^11^ ^,^ ^28^ ^,^ ^33^ and geographic region ^15^ ^,^ ^28^ on the temperature-mortality association. Thus, the annual average of mean daily temperatures, annual mean temperature range, daily amplitude of temperature (difference between maximum and minimum daily temperature), latitude and geographic region were included in the model as geographic factors modifying the temperature-mortality association.

### Urban and socioeconomic data

Individual and community characteristics were identified as modifying factors of the temperature-mortality association ^17^. Among the factors at the individual level we have schooling ^7^ ^,^ ^11^ ^,^ ^32^ and income ^11^ ^,^ ^18^ ^,^ ^34^. Population density ^8^ ^,^ ^18^ ^,^ ^32^ ^,^ ^35^ and GDP per capita ^18^ ^,^ ^32^ are community factors that characterize the level of urban development.

Hence, socioeconomic and urban development data were included in the model as modifying factors for the temperature-mortality association. These data were obtained from the 2010 Demographic Census (https://censo2010.ibge.gov.br/) by the Brazilian Institute of Geography and Statistics (IBGE, acronym in Portuguese) for each municipality, and then the respective metropolitan area averages were calculated.

Urban indicators used were demographic density (inhabitants/km^2^) and GDP per capita (BRL). Socioeconomic indicators used were income (percentage of individuals with no income or with an income of up to one minimum wage) and schooling (percentage of individuals over 10 years old with complete primary education). For each metropolitan area we estimated the annual average of each numerical variable and the average for the period studied, which was the value used in the analyses.

### Data analysis

Two-step analysis investigated the temperature-mortality association separately for each group of mortality causes.

Firstly, a time series analysis was performed for each metropolitan area and group using the generalized additive model (GAM), assuming a quasi-Poisson model ^36^. A cross-basis function of the distributed lag non-linear models (DLNM) ^37^ was included to model the non-linear lagged effect of ambient temperature on mortality. This function was defined by cubic natural spline with three internal nodes placed on the 10th, 75th, and 90th percentiles of each site’s specific temperature distribution, and a cubic natural spline with nodes arranged at the intercept and three equally spaced internal nodes in the log scale of lag values. A 21-day analysis window (maximum lag up to 21 days) was used, allowing us to estimate the lagged relation between temperature and mortality, less effect of death anticipation, and to compare our results with previous studies.

Two thin plate splines were included on the regression model, one for adjusting the time and seasonal trend, and one for relative air humidity. Time trend and seasonality were adjusted using splines from 2 to 8 df and the choice was based on the Akaike information criterion (AIC) and analysis of residuals. Relative humidity adjustments tested 3 to 6 df, and the choice was made based on the lowest AIC. Finally, an indicator variable was included for each day of the week.

Based on this model, the minimum mortality percentile (MMP) and the respective minimum mortality temperature (MMT) were estimated for each metropolitan area and group of causes ^37^. The effect of temperature on mortality was estimated in relative risk (RR). The effect of cold was estimated by the RR of mortality between the 1st percentile and MMP, and the effect of heat was estimated from the RR of mortality between the 99th percentile and MMP. Confidence intervals (95%CI) were extracted from these values using a 95% confidence level.

Secondly, the degree of heterogeneity between locations was verified and the mean value of the temperature-mortality association was estimated for the entire country, for the geographic regions and for each group of causes. Using the same regression model described above, the entire temperature-mortality association accumulated in the lag period was reduced by extracting the vectors of the estimated coefficients and the respective matrix of estimated (co)variances for each location and group. This step reduces the number of parameters considered in the second-stage meta-analysis while preserving the complexity of the estimated dependency ^26^. Mean value of the MMP estimated in the individual analyses was chosen as the reference for the estimates.

A multivariate meta-analysis model ^26^ defined the mean temperature-mortality association of the metropolitan areas at the national level and for each Brazilian region using the restricted maximum likelihood (REML) method. Quantification of heterogeneity in the exposure-response relations of the metropolitan areas used the Cochran Q test for (residual) heterogeneity and I^2^ statistics.

Univariable multivariate meta-regression models evaluated the modifying effect attributable to the following metapredictive variables: mean, daily and annual amplitude of mean temperature, latitude, demographic density, GDP per capita, schooling level and income. Meta-regression models, each including a single metapredictor, were specified and exposure-response associations were estimated for the 25th and 75th percentiles values of these metapredictor variables. Each model was tested for heterogeneity (Q-test and I^2^) and model fit (AIC). Wald test assessed the significance of the multivariate association between the outcome parameters and each predictor variable.

Sensitivity analysis was performed by testing different parameters for cross-basis. Two spline functions (natural cubic spline [ns] and quadratic B-spline [bs]) were tested, and two distributions for internal knots (10th, 75th and 90th percentiles and 25th, 50th and 75th percentiles) of the temperature distribution. Using the Q-AIC, an AIC modified for likelihood models ^38^, the model with the lowest value in the sum of Q-AICs of all metropolitan areas was chosen as the best fit.

All statistical analyses and graphs were performed on the R platform version 3.5.1 using *dlnm*, *mgcv* and *mvmeta*.

## Results

A total of 6,483,270 deaths from non-accidental causes occurred between 2000 and 2014 in the 42 metropolitan areas analyzed, of which 4,290,322 were individuals over 60 years old. Table 1 summarizes mortality and climate data for each location. Metropolitan areas extend from latitude 2º82’ North (Metropolitan Area of Capital/Roraima State) to 30º5’ South (Metropolitan Area of Porto Alegre/Rio Grande do Sul State), with mean ambient temperature ranging from 14.87ºC (Metropolitan Area of Lages/Santa Catarina State) to 28.11ºC (Metropolitan Area of Capital/Roraima State).

**Table 1:** Data summary on total deaths by group and climatic data in the metropolitan areas from 2000 to 2014, Brazil.

Metropolitan area	Sum of deaths					Climate data			
	General	Older adults				Ambiente temperature (ºC)		**Average humidity *lag01* (%)**	
	Non-accidental	Non-accidental	Circulatory	Respiratory	Other causes	Mean	SD	Mean	SD
Central-West									
Goiânia	131,706	82,553	31,584	14,588	36,381	24.56	1.98	60.74	15.29
Vale do Rio Cuiabá	60,040	35,572	13,575	4,980	17,017	26.44	2.75	72.20	11.20
Northeast									
Aracaju	50,540	30,876	10,826	3,849	16,201	26.49	1.20	76.92	4.78
Campina Grande	47,411	33,459	10,616	2,598	20,245	23.50	1.43	78.58	7.07
Cariri	39,766	27,367	10,228	3,429	13,710	25.98	1.62	70.05	13.28
Feira de Santana	50,030	32,554	11,732	3,251	17,571	24.39	2.04	80.49	8.74
Fortaleza	225,488	147,691	48,628	19,978	79,085	27.02	0.90	77.42	6.04
Grande São Luís	80,099	47,464	18,096	5,036	24,332	26.96	0.94	82.48	5.47
João Pessoa	81,534	54,591	21,347	6,870	26,374	26.97	1.33	76.73	6.11
Maceió	83,503	49,992	19,760	6,749	23,483	25.28	1.36	79.68	5.62
Natal	83,969	56,514	20,619	6,646	29,249	26.51	1.26	80.52	4.75
Palmeira dos Índios	9,378	6,644	1,979	802	3,863	24.72	1.94	75.47	9.58
Patos	15,202	11,159	4,320	1,128	5,711	27.64	1.56	59.86	11.75
Recife	295,368	193,198	78,787	30,639	83,772	25.97	1.34	77.80	6.79
Salvador	227,941	134,597	48,482	18,918	67,197	25.51	1.55	81.80	5.32
Sudoeste Maranhense	20,227	12,141	4,163	1,125	6,853	27.78	1.45	72.63	11.07
North									
Belém	141,727	85,631	29,064	14,623	41,944	26.90	0.87	83.41	5.68
Capital	13,217	6,764	2,419	852	3,493	28.11	1.39	73.90	9.63
Gurupi	8,379	5,511	2,481	618	2,412	26.15	1.66	69.01	14.66
Macapá	20,592	10,392	3,268	1,377	5,747	27.44	1.24	79.99	7.48
Manaus	112,740	62,332	16,763	7,445	38,124	27.36	1.41	82.33	6.51
Palmas	15,857	9,557	4,016	1,191	4,350	27.19	1.64	67.87	15.47
Southeast									
Belo Horizonte	365,476	236,525	86,501	34,075	115,949	21.84	2.41	64.73	11.30
Grande Vitória	107,848	68,127	28,947	8,009	31,171	24.82	2.36	76.30	6.37
Ribeirão Preto	118,609	84,466	31,641	13,009	39,816	22.45	3.01	68.32	12.43
Rio de Janeiro	1,206,529	832,277	295,955	118,357	417,965	25.22	3.11	71.93	7.52
São Paulo	1,492,580	983,815	403,501	155,806	424,508	20.55	3.40	73.56	8.99
Sorocaba	145,450	100,193	33,220	16,073	50,900	21.21	3.33	74.33	8.64
Vale do Aço	46,841	31,130	10,581	4,402	16,147	21.68	2.63	75.90	8.78
Vale do Paraíba e Litoral Norte	171,065	113,572	37,819	17,035	58,718	20.62	3.36	77.67	7.08
South									
Campo Mourão	26,374	19,494	9,010	2,982	7,502	20.36	3.91	83.24	9.18
Carbonífera	35,315	24,259	10,142	3,223	10,894	19.81	4.46	83.71	6.90
Chapecó	25,519	18,547	6,708	3,001	8,838	19.43	4.73	72.86	11.88
Contestado	31,901	22,956	7,621	3,422	11,913	16.77	4.40	76.29	11.42
Curitiba	221,955	147,246	57,287	20,975	68,984	17.82	3.85	80.43	8.10
Florianópolis	59,215	40,232	16,477	5,391	18,364	21.19	3.77	79.05	6.89
Lages	28,541	19,439	6,458	2,917	10,064	14.87	4.27	81.49	8.52
Londrina	78,668	56,761	22,559	8,884	25,318	21.89	3.75	74.30	10.88
Maringá	49,727	36,322	14,538	5,273	16,511	22.57	3.73	68.91	13.13
Porto Alegre	364,965	251,881	93,795	38,466	119,620	19.86	4.87	76.84	8.69
Serra Gaúcha	49,351	36,088	13,012	4,901	18,175	17.23	4.81	76.94	10.65
Vale do Itajaí	42,627	30,433	11,752	4,058	14,623	20.87	3.99	85.95	7.02

SD: standard deviation.

Non-accidental temperature-mortality association accumulated in 21 days was estimated for the General and Older Adult groups. General group presented an estimated RR for the effect of heat of 1.09 (95%CI: 1.04-1.15) and for cold of 1.26 (95%CI: 1.21-1.32). In the Older Adult group, the estimated RR for the effect of heat was 1.13 (95%CI: 1.07-1.20) and for cold was 1.30 (95%CI: 1.24-1.36). We observed a greater effect of cold than heat in both groups. These values result from combining the estimated RR for each of the 42 Brazilian metropolitan areas.

### Variability of the temperature-mortality association across the Brazilian territory

**Figure 1:**
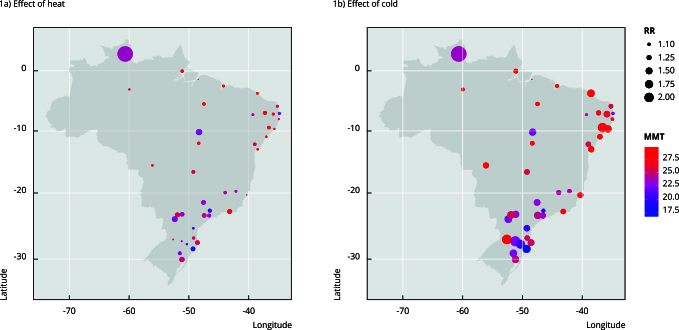
Geographic distribution of the non-accidental temperature-mortality association estimates in the Older Adult group.

MMT: minimum mortality temperature; RR: relative risk.

In observing the estimated values for each metropolitan area (Table 2), we note a significant increase of RR associated with extreme temperatures (both high and low) in non-accidental mortality for the General and Older Adult groups in several locations, especially in South and Southeast Brazil. Most metropolitan areas had higher RRs estimated for the effect of cold. RR values of the temperature-mortality association are higher in the Older Adult group than in the General group for most locations. Figure 1 illustrates the variability in RR results between metropolitan areas throughout the national territory, using the RR estimates of the Older Adult group as an example.

**Table 2:** Estimates of the association of extreme high (99th percentile x minimum mortality percentile [MMP]) and low (1st percentile x MMP) temperatures in cumulative non-accidental mortality over a 21-day period for each group and location.

Metropolitan area	General group				Older Adulst group			
	MMT	MMP	RR (95%CI) - heat	RR (95%CI) - cold	MMT	MMP	RR (95%CI) - heat	RR (95%CI) - cold
Central-West								
Goiânia	24.50	0.50	1.16 (1.08-1.24)	1.25 (1.14-1.37)	25.90	0.77	1.23 (1.13-1.34)	1.32 (1.19-1.46)
Vale do Rio Cuiabá	33.40	1.00	1.06 (0.76-1.47)	1.53 (0.93-2.54)	29.70	0.92	1.07 (0.85-1.34)	1.38 (1.04-1.84)
Northeast								
Aracaju	29.90	1.00	1.03 (0.70-1.53)	1.21 (0.75-1.95)	29.90	1.00	1.07 (0.65-1.78)	1.30 (0.70-2.40)
Campina Grande	26.80	1.00	1.14 (0.96-1.34)	1.55 (1.17-2.04)	26.80	1.00	1.12 (0.92-1.36)	1.46 (1.06-2.01)
Cariri	31.20	1.00	1.07 (0.66-1.75)	1.17 (0.60-2.26)	24.30	0.13	1.10 (0.86-1.42)	1.07 (0.89-1.28)
Feira de Santana	26.40	0.82	1.22 (1.07-1.39)	1.29 (1.09-1.53)	25.80	0.71	1.19 (1.02-1.40)	1.30 (1.07-1.58)
Fortaleza	29.60	1.00	1.11 (0.93-1.31)	1.71 (1.31-2.22)	29.60	1.00	1.09 (0.94-1.26)	1.62 (1.27-2.06)
Grande São Luís	23.00	0.00	1.31 (0.71-2.40)	1.20 (0.78-1.84)	28.10	0.90	1.11 (0.94-1.31)	1.14 (0.87-1.48)
João Pessoa	30.30	1.00	1.09 (0.86-1.37)	1.12 (0.84-1.49)	22.20	0.00	1.14 (0.71-1.83)	1.10 (0.80-1.5)
Maceió	27.60	0.97	1.00 (0.92-1.09)	1.31 (1.01-1.68)	28.70	1.00	1.05 (0.74-1.49)	1.58 (0.99-2.53)
Natal	25.50	0.23	1.12 (0.99-1.27)	1.23 (1.04-1.45)	25.90	0.31	1.12 (0.97-1.30)	1.22 (1.00-1.48)
Palmeira dos Índios	27.20	0.90	1.34 (0.90-2.00)	1.64 (1.14-2.34)	27.40	0.92	1.20 (0.76-1.90)	2.01 (1.31-3.1)
Patos	27.40	0.45	1.15 (0.86-1.54)	1.11 (0.87-1.42)	27.30	0.43	1.28 (0.89-1.84)	1.26 (0.92-1.74)
Recife	29.00	1.00	1.01 (0.93-1.11)	1.11 (0.95-1.31)	27.50	0.88	1.04 (0.96-1.13)	1.11 (0.99-1.24)
Salvador	29.10	1.00	1.08 (0.99-1.18)	1.37 (1.19-1.57)	29.10	1.00	1.07 (0.97-1.19)	1.40 (1.19-1.66)
Sudoeste Maranhense	29.20	0.83	1.03 (0.80-1.32)	1.15 (0.76-1.72)	29.40	0.86	1.18 (0.85-1.64)	1.18 (0.69-2.00)
North								
Belém	31.40	1.00	1.22 (0.35-4.29)	1.33 (0.35-5.00)	28.20	0.95	1.01 (0.91-1.11)	1.02 (0.87-1.20)
Capital	32.80	1.00	2.01 (0.14-29.03)	1.72 (0.10-30.20)	23.40	0.00	7.21 (1.26-41.16)	4.03 (1.37-11.8)
Gurupi	32.20	1.00	1.38 (0.41-4.64)	1.70 (0.39-7.35)	27.60	0.81	1.16 (0.77-1.76)	1.21 (0.91-1.60)
Macapá	30.70	1.00	1.07 (0.44-2.61)	1.20 (0.41-3.54)	28.10	0.65	1.17 (0.88-1.57)	1.23 (0.88-1.71)
Manaus	29.10	0.88	1.12 (1.01-1.24)	1.10 (0.99-1.23)	27.60	0.57	1.05 (0.92-1.20)	1.12 (0.97-1.28)
Palmas	29.50	0.90	1.29 (0.92-1.82)	1.06 (0.64-1.76)	22.10	0.00	1.79 (0.41-7.85)	1.56 (0.58-4.17)
Southeast								
Belo Horizonte	22.10	0.50	1.15 (1.09-1.21)	1.14 (1.09-1.20)	24.00	0.80	1.18 (1.11-1.25)	1.26 (1.20-1.33)
Grande Vitória	31.40	1.00	1.04 (0.60-1.81)	1.44 (0.76-2.73)	27.30	0.82	1.01 (0.86-1.20)	1.39 (1.20-1.61)
Ribeirão Preto	22.60	0.44	1.32 (1.20-1.45)	1.39 (1.24-1.56)	23.10	0.50	1.41 (1.26-1.56)	1.47 (1.28-1.68)
Rio de Janeiro	26.60	0.67	1.34 (1.29-1.40)	1.27 (1.23-1.32)	27.00	0.71	1.45 (1.38-1.51)	1.31 (1.26-1.37)
São Paulo	23.50	0.79	1.19 (1.16-1.23)	1.36 (1.32-1.40)	23.50	0.79	1.26 (1.21-1.31)	1.41 (1.35-1.46)
Sorocaba	24.80	0.86	1.30 (1.19-1.42)	1.62 (1.45-1.82)	24.30	0.81	1.39 (1.25-1.55)	1.60 (1.40-1.82)
Vale do Aço	24.90	0.91	1.13 (0.95-1.35)	1.20 (1.01-1.42)	24.10	0.81	1.11 (0.90-1.37)	1.21 (1.00-1.45)
Vale do Paraíba e Litoral Norte	20.20	0.43	1.21 (0.94-1.55)	1.23 (1.02-1.49)	19.70	0.39	1.28 (0.92-1.76)	1.16 (0.92-1.46)
South								
Campo Mourão	21.90	0.57	1.73 (1.28-2.33)	1.59 (1.29-1.95)	22.10	0.59	1.66 (1.16-2.37)	1.63 (1.28-2.08)
Carbonífera	17.90	0.33	1.26 (1.07-1.49)	1.69 (1.44-1.99)	16.70	0.25	1.40 (1.13-1.72)	1.74 (1.42-2.12)
Chapecó	24.90	0.92	1.06 (0.83-1.36)	2.05 (1.59-2.63)	28.90	1.00	1.02 (0.72-1.43)	2.05 (1.11-3.81)
Contestado	21.80	0.90	1.13 (0.91-1.40)	1.94 (1.55-2.42)	22.50	0.94	1.03 (0.83-1.27)	2.16 (1.68-2.80)
Curitiba	19.80	0.66	1.09 (1.01-1.16)	1.42 (1.32-1.52)	20.10	0.69	1.08 (0.99-1.17)	1.44 (1.32-1.57)
Florianópolis	25.10	0.83	1.38 (1.23-1.55)	1.30 (1.11-1.53)	25.20	0.84	1.41 (1.22-1.62)	1.49 (1.23-1.81)
Lages	20.20	0.92	1.05 (0.89-1.24)	1.72 (1.37-2.16)	20.30	0.93	1.06 (0.87-1.29)	1.91 (1.45-2.51)
Londrina	23.20	0.57	1.26 (1.13-1.41)	1.51 (1.33-1.70)	23.40	0.59	1.38 (1.20-1.58)	1.55 (1.34-1.80)
Maringá	24.00	0.59	1.26 (1.09-1.45)	1.51 (1.33-1.71)	25.50	0.79	1.46 (1.22-1.74)	1.59 (1.36-1.86)
Porto Alegre	24.70	0.83	1.45 (1.37-1.54)	1.53 (1.42-1.65)	24.80	0.84	1.55 (1.45-1.66)	1.57 (1.44-1.72)
Serra Gaúcha	22.40	0.87	1.23 (1.08-1.41)	1.52 (1.27-1.81)	22.50	0.88	1.24 (1.05-1.46)	1.61 (1.29-2.01)
Vale do Itajaí	18.60	0.28	1.11 (0.91-1.37)	1.30 (1.06-1.59)	25.30	0.87	1.15 (0.97-1.35)	1.29 (0.96-1.72)

95%CI: 95% confidence interval; MMT: minimum mortality temperature; RR: relative risk.

Nota: in bold are the significant RR values.

We also note that the MMT also varies between metropolitan areas. MMT is the optimal ^2^, most comfortable or ideal temperature from which mortality increases ^38^. Estimated MMT values ranged from 17.9 to 33.4ºC for the General group (mean: 26.1ºC, standard deviation - SD: 4.1) and from 16.7 to 29.9ºC for the Older Adult group (mean: 25.4ºC, SD: 3.1), with higher MMT values found in latitudes close to the Equator and lower in metropolises located further south.

**Figure 2:**
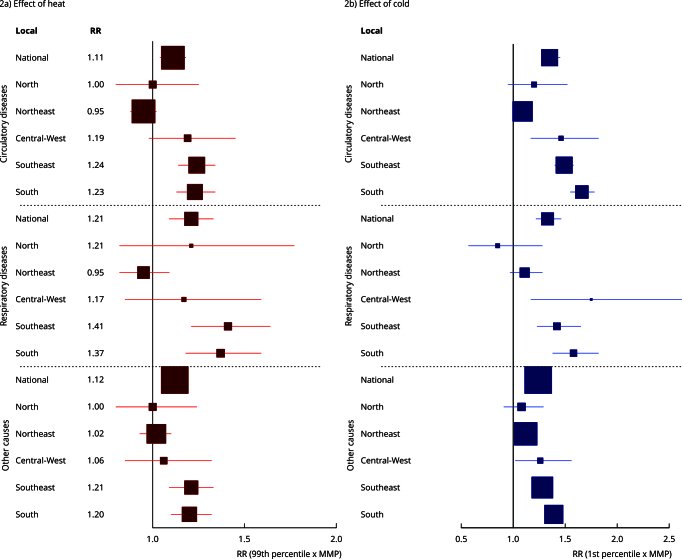
Mean estimates of the cumulative effect of ambient temperature on the mortality over a 21-day period of the Older Adult subgroups for Brazil and each region.

Tables 3, 4, and 5 present the effects of extreme temperatures on circulatory, respiratory and other mortality causes in the Older Adult group by metropolitan area. Figure 2 shows the estimated effect (RR) of high and low temperatures on Older Adult subgroups’ mortality by geographic region. Differences in RR can be noted by geographic region and cause. Cold and heat effect was significant on the mortality of the three Older Adult subgroups in the South and Southeast regions. In the Central-West, only cold affected mortality. Northeast locations had only RR of circulatory mortality and other causes associated with extreme low temperatures. Northern metropolitan areas had no significant RRs.

**Table 3:** Estimates of the association of extreme high (99th percentile x minimum mortality percentile [MMP]) and low (1st percentile x MMP) temperatures in cumulative circulatory mortality over a 21-day period for each Older Adult group and location..

Metropolitan area	Circulatory mortality - Older Adult group			
	MMT	MMP	RR (95%CI) - heat	RR (95%CI) - cold
Central-West				
Goiânia	26.60	0.85	1.16 (1.01-1.34)	1.38 (1.18-1.61)
Vale do Rio Cuiabá	29.30	0.90	1.26 (0.87-1.82)	1.47 (0.95-2.28)
Northeast				
Aracaju	29.90	1.00	2.16 (0.99-4.71)	2.15 (0.84-5.50)
Campina Grande	26.80	1.00	1.62 (1.16-2.27)	1.79 (1.03-3.13)
Cariri	31.20	1.00	1.96 (0.76-5.07)	2.51 (0.68-9.30)
Feira de Santana	26.10	0.77	1.09 (0.84-1.41)	1.55 (1.11-2.16)
Fortaleza	26.00	0.13	1.04 (0.90-1.21)	1.27 (1.00-1.62)
Grande São Luís	26.00	0.16	1.16 (0.86-1.56)	1.43 (0.88-2.31)
João Pessoa	30.30	1.00	1.66 (1.05-2.63)	1.59 (0.90-2.80)
Maceió	28.70	1.00	1.25 (0.70-2.23)	2.61 (1.22-5.61)
Natal	25.90	0.31	1.04 (0.85-1.27)	1.49 (1.09-2.02)
Patos	27.10	0.39	1.20 (0.77-1.85)	1.32 (0.85-2.04)
Recife	27.80	0.94	1.01 (0.91-1.12)	1.05 (0.88-1.25)
Salvador	29.10	1.00	1.14 (0.96-1.35)	1.43 (1.09-1.87)
Sudoeste Maranhense	29.60	0.89	1.63 (0.98-2.71)	1.05 (0.47-2.35)
North				
Belém	26.00	0.15	1.08 (0.88-1.32)	1.14 (0.86-1.50)
Macapá	22.90	0.00	1.84 (0.29-11.52)	1.44 (0.36-5.74)
Manaus	30.20	0.97	1.00 (0.88-1.15)	1.44 (1.05-1.98)
Southeast				
Belo Horizonte	24.10	0.82	1.18 (1.06-1.32)	1.34 (1.23-1.46)
Grande Vitória	27.70	0.88	1.12 (0.87-1.43)	1.51 (1.19-1.92)
Ribeirão Preto	24.60	0.75	1.15 (1.01-1.31)	1.48 (1.24-1.75)
Rio de Janeiro	28.20	0.81	1.40 (1.29-1.52)	1.46 (1.35-1.57)
São Paulo	24.20	0.85	1.23 (1.15-1.30)	1.66 (1.55-1.77)
Sorocaba	23.50	0.73	1.50 (1.29-1.74)	1.50 (1.27-1.79)
Vale do Aço	24.50	0.86	1.21 (0.85-1.73)	1.42 (1.05-1.92)
Vale do Paraíba e Litoral Norte	28.30	1.00	1.38 (0.70-2.74)	1.69 (0.60-4.80)
South				
Campo Mourão	22.00	0.58	1.35 (0.83-2.18)	1.78 (1.32-2.40)
Carbonífera	25.00	0.88	1.24 (0.95-1.63)	1.61 (1.08-2.39)
Chapecó	25.00	0.92	1.10 (0.68-1.78)	2.44 (1.50-3.98)
Contestado	22.80	0.95	1.02 (0.73-1.43)	2.21 (1.43-3.43)
Curitiba	20.20	0.70	1.05 (0.91-1.21)	1.72 (1.50-1.97)
Florianópolis	25.60	0.87	1.21 (0.96-1.54)	2.06 (1.50-2.83)
Lages	20.60	0.95	1.07 (0.78-1.46)	2.65 (1.64-4.27)
Londrina	23.70	0.62	1.26 (1.02-1.56)	1.86 (1.53-2.26)
Maringá	26.30	0.88	1.51 (1.12-2.03)	1.67 (1.29-2.16)
Porto Alegre	24.70	0.83	1.48 (1.33-1.64)	1.86 (1.62-2.13)
Serra Gaúcha	21.50	0.80	1.42 (1.10-1.84)	1.36 (1.00-1.87)
Vale do Itajaí	17.70	0.21	1.57 (1.17-2.12)	1.69 (1.28-2.22)

95%CI: 95% confidence interval; MMT: minimum mortality temperature; RR: relative risk.

Nota: in bold are the significant RR values.

**Table 4:** Estimates of the association of extreme high (99th percentile x minimum mortality percentile [MMP]) and low (1st percentile x MMP) temperatures in cumulative respiratory mortality over a 21-day period for each Older Adult group and location.

Metropolitan area	Respiratory mortality - Older Adult group			
	MMT	MMP	RR (95%CI) - heat	RR (95%CI) - cold
Central-West				
Goiânia	25.10	0.63	1.34 (1.13-1.61)	1.54 (1.21-1.97)
Vale do Rio Cuiabá	33.40	1.00	1.38 (0.49-3.91)	2.72 (0.59-12.50)
Northeast				
Aracaju	29.90	1.00	4.65 (1.22-17.71)	5.12 (1.02-25.66)
Campina Grande	25.30	0.92	1.17 (0.76-1.80)	1.73 (0.88-3.39)
Cariri	24.30	0.13	1.28 (0.63-2.60)	1.07 (0.69-1.65)
Feira de Santana	27.00	0.91	1.03 (0.64-1.65)	1.61 (0.88-2.95)
Fortaleza	28.70	0.98	1.00 (0.92-1.09)	1.70 (1.15-2.52)
Grande São Luís	23.00	0.00	2.34 (0.19-29.01)	1.41 (0.24-8.20)
João Pessoa	25.20	0.12	1.24 (0.88-1.74)	1.13 (0.69-1.84)
Maceió	28.70	1.00	2.22 (0.82-5.98)	1.88 (0.51-6.93)
Natal	21.80	0.00	2.59 (0.44-15.08)	1.66 (0.51-5.41)
Patos	23.20	0.00	2.17 (0.27-17.77)	1.77 (0.58-5.42)
Recife	26.20	0.52	1.10 (0.91-1.32)	1.29 (1.01-1.64)
Salvador	29.10	1.00	1.14 (0.87-1.49)	2.09 (1.37-3.21)
Sudoeste Maranhense	26.40	0.17	1.25 (0.49-3.17)	3.95 (0.86-18.22)
North				
Belém	23.00	0.00	1.23 (0.29-5.23)	1.05 (0.36-3.10)
Macapá	22.90	0.00	3.86 (0.24-62.9)	3.17 (0.4-25.46)
Manaus	21.90	0.00	4.87 (0.73-32.41)	2.78 (0.61-12.70)
Southeast				
Belo Horizonte	23.30	0.69	1.33 (1.14-1.56)	1.29 (1.14-1.45)
Grande Vitória	27.10	0.80	1.20 (0.73-1.95)	1.62 (1.07-2.44)
Ribeirão Preto	23.10	0.50	1.58 (1.27-1.96)	2.03 (1.57-2.61)
Rio de Janeiro	23.60	0.32	1.73 (1.52-1.97)	1.27 (1.11-1.45)
São Paulo	22.20	0.65	1.49 (1.36-1.63)	1.28 (1.18-1.40)
Sorocaba	18.40	0.20	1.61 (1.20-2.17)	1.42 (1.04-1.93)
Vale do Aço	21.50	0.44	1.18 (0.75-1.86)	1.82 (1.31-2.54)
Vale do Paraíba e Litoral Norte	21.10	0.51	1.50 (0.80-2.79)	1.25 (0.83-1.90)
South				
Campo Mourão	21.20	0.50	1.79 (0.75-4.31)	1.99 (1.20-3.29)
Carbonífera	16.10	0.21	2.13 (1.16-3.91)	2.89 (1.74-4.78)
Chapecó	28.90	1.00	1.57 (0.63-3.95)	4.54 (0.87-23.78)
Contestado	21.40	0.87	1.36 (0.67-2.75)	2.38 (1.26-4.50)
Curitiba	3.30	0.00	1.29 (0.70-2.36)	1.25 (0.84-1.87)
Florianópolis	16.70	0.13	1.79 (1.08-2.97)	1.13 (0.76-1.68)
Lages	10.20	0.14	1.24 (0.68-2.27)	3.22 (1.88-5.52)
Londrina	25.00	0.79	1.55 (1.10-2.19)	1.46 (0.99-2.15)
Maringá	23.30	0.51	1.24 (0.80-1.91)	2.39 (1.65-3.45)
Porto Alegre	24.30	0.80	1.90 (1.61-2.23)	1.60 (1.31-1.95)
Serra Gaúcha	29.70	1.00	1.16 (0.33-4.07)	2.52 (0.51-12.34)
Vale do Itajaí	17.50	0.20	1.63 (0.97-2.74)	1.47 (0.91-2.36)

95%CI: 95% confidence interval; MMT: minimum mortality temperature; RR: relative risk.

Nota: in bold are the significant RR values.

**Table 5:** Estimates of the association of extreme high (99th percentile x minimum mortality percentile [MMP]) and low (1st percentile x MMP) temperatures in cumulative other causes mortality over a 21-day period for each Older Adult group and location.

Metropolitan area	Other causes mortality - Older Adult group			
	MMT	MMP	RR (95%CI) - heat	RR (95%CI) - cold
Central-West				
Goiânia	22.70	0.16	1.15 (1.03-1.29)	1.20 (1.01-1.42)
Vale do Rio Cuiabá	31.20	0.99	1.00 (0.93-1.08)	1.34 (0.86-2.08)
Northeast				
Aracaju	28.00	0.91	1.06 (0.90-1.26)	1.34 (1.06-1.69)
Campina Grande	25.80	0.97	1.01 (0.92-1.10)	1.40 (1.08-1.82)
Cariri	21.20	0.00	1.66 (0.81-3.44)	1.26 (0.77-2.06)
Feira de Santana	18.50	0.00	1.25 (0.71-2.21)	1.33 (0.93-1.90)
Fortaleza	29.60	1.00	1.16 (0.95-1.42)	1.83 (1.32-2.54)
Grande São Luís	23.00	0.00	1.18 (0.39-3.51)	1.17 (0.55-2.51)
João Pessoa	22.20	0.00	1.16 (0.59-2.27)	1.13 (0.73-1.77)
Maceió	27.00	0.90	1.12 (0.86-1.45)	1.13 (0.82-1.56)
Natal	26.10	0.35	1.14 (0.97-1.35)	1.11 (0.86-1.43)
Patos	31.80	1.00	1.46 (0.57-3.75)	1.68 (0.40-6.96)
Recife	27.70	0.92	1.04 (0.95-1.13)	1.18 (1.04-1.34)
Salvador	29.10	1.00	1.01 (0.88-1.17)	1.24 (0.98-1.56)
Sudoeste Maranhense	22.40	0.00	1.46 (0.25-8.42)	1.32 (0.39-4.47)
North				
Belém	31.40	1.00	1.47 (0.17-13.08)	1.57 (0.16-15.49)
Macapá	28.80	0.85	1.02 (0.68-1.54)	1.38 (0.88-2.17)
Manaus	28.00	0.68	1.07 (0.90-1.26)	1.10 (0.93-1.31)
Southeast				
Belo Horizonte	23.90	0.79	1.13 (1.04-1.24)	1.20 (1.12-1.29)
Grande Vitória	31.40	1.00	1.23 (0.46-3.30)	1.59 (0.50-5.10)
Ribeirão Preto	22.80	0.47	1.38 (1.21-1.57)	1.28 (1.07-1.53)
Rio de Janeiro	26.00	0.61	1.41 (1.32-1.51)	1.28 (1.21-1.37)
São Paulo	22.20	0.65	1.23 (1.17-1.29)	1.26 (1.19-1.33)
Sorocaba	24.70	0.86	1.30 (1.15-1.47)	1.65 (1.38-1.98)
Vale do Aço	28.50	1.00	1.23 (0.70-2.17)	1.66 (0.78-3.53)
Vale do Paraíba e Litoral Norte	20.40	0.45	1.36 (0.97-1.91)	1.29 (1.02-1.65)
South				
Campo Mourão	23.30	0.75	1.65 (0.95-2.87)	1.96 (1.34-2.88)
Carbonífera	18.40	0.37	1.22 (0.96-1.55)	1.72 (1.31-2.27)
Chapecó	28.90	1.00	1.12 (0.70-1.78)	1.88 (0.82-4.28)
Contestado	26.50	1.00	1.04 (0.54-2.00)	2.12 (0.82-5.44)
Curitiba	22.30	0.88	1.08 (0.96-1.21)	1.38 (1.20-1.59)
Florianópolis	25.20	0.84	1.52 (1.25-1.87)	1.22 (0.92-1.63)
Lages	17.10	0.66	1.04 (0.81-1.34)	1.31 (0.96-1.81)
Londrina	22.90	0.54	1.47 (1.22-1.77)	1.59 (1.32-1.91)
Maringá	24.20	0.62	1.51 (1.19-1.91)	1.37 (1.10-1.72)
Porto Alegre	25.10	0.86	1.46 (1.34-1.59)	1.38 (1.21-1.57)
Serra Gaúcha	22.80	0.90	1.20 (0.99-1.46)	1.81 (1.41-2.33)
Vale do Itajaí	17.70	0.21	1.18 (0.91-1.53)	1.41 (1.06-1.86)

95%CI: 95% confidence interval; MMT: minimum mortality temperature; RR: relative risk.

Nota: in bold are the significant RR values.

95%CI: 95% confidence interval; MMP: minimum mortality percentile; RR: relative risk. Nota: square marks the estimated RR and the horizontal line marks the 95%CI. Square size is the inverse of the 95%CI range.

Variability in the estimates of temperature-mortality associations between metropolitan areas was tested using the heterogeneity test (I^2^). Association between temperature and non-accidental mortality showed I^2^ values of 81% in the General group and 79% in the Older Adult group. Heterogeneity analyses found lower I^2^ values for the Older Adult subgroups. Including the geographic region factor into the model reduced the I^2^ values for all groups (Table 6).

**Table 6:** Results of the Cochran Q test, I^2^ statistics, Akaike information criterion (AIC), and Wald test for meta-regression models for each group.

Metapredictor	I2	Q test (p-value)	AIC	Wald test (p-value)
General group				
Non-accidental mortality				
None	81.00	0.00000	26.81	0.00000
Region	65.10	0.00000	54.75	0.00000
Geographic factors				
Latitude	67.10	0.00000	31.65	0.00000
Annual mean temperature range	64.70	0.00000	18.39	0.00000
Daily mean temperature range	77.00	0.00000	49.89	0.01300
Average mean temperature	75.90	0.00000	36.64	0.00000
Urban factors				
Population density	80.50	0.00000	107.77	0.85977
GDP per capita	80.70	0.00000	121.77	0.18142
Socioeconomic factors				
Schooling	73.10	0.00000	42.46	0.00004
Income	69.10	0.00000	45.44	0.00000
Older Adult group				
Non-accidental mortality				
None	79.00	0.00000	56.76	0.00000
Region	60.00	0.00000	79.33	0.00000
Geographic factors				
Latitude	62.90	0.00000	58.43	0.00000
Annual mean temperature range	61.20	0.00000	49.57	0.00000
Daily mean temperature range	73.10	0.00000	74.49	0.00033
Average mean temperature	74.00	0.00000	63.13	0.00000
Urban factors				
Population density	78.30	0.00000	138.03	0.80990
GDP per capita	78.60	0.00000	149.71	0.06470
Socioeconomic factors				
Schooling	70.20	0.00000	71.91	0.00002
Income	65.20	0.00000	71.35	0.00000
Circulatory mortality				
None	68.80	0.00000	116.89	0.00000
Region	40.60	0.00000	128.29	0.00000
Geographic factors				
Latitude	40.50	0.00000	116.67	0.00000
Annual mean temperature range	39.60	0.00000	112.29	0.00000
Daily mean temperature range	60.40	0.00000	135.82	0.00364
Average mean temperature	54.90	0.00000	122.86	0.00000
Urban factors				
Population density	68.10	0.00000	194.42	0.52725
GDP per capita	67.10	0.00000	206.29	0.03023
Socioeconomic factors				
Schooling	58.80	0.00000	123.70	0.00000
Income	46.80	0.00000	131.34	0.00000
Respiratory mortality				
None	59.10	0.00000	253.10	0.00000
Region	39.60	0.00000	263.74	0.00000
Geographic factors				
Latitude	45.30	0.00000	268.79	0.00000
Annual mean temperature range	42.30	0.00000	262.80	0.00000
Daily mean temperature range	48.40	0.00000	263.54	0.00001
Average mean temperature	52.80	0.00000	268.66	0.00086
Urban factors				
Population density	57.70	0.00000	326.94	0.54401
GDP per capita	59.00	0.00000	340.70	0.12657
Socioeconomic factors				
Schooling	50.30	0.00000	267.58	0.00349
Income	45.70	0.00000	272.33	0.00000
Other causes				
None	63.90	0.00000	56.83	0.00000
Region	42.80	0.00000	85.49	0.00000
Geographic factors				
Latitude	45.70	0.00000	72.29	0.00000
Annual mean temperature range	45.40	0.00000	67.81	0.00000
Daily mean temperature range	56.70	0.00000	76.90	0.00488
Average mean temperature	59.60	0.00000	74.68	0.00014
Urban factors				
Population density	63.50	0.00000	134.38	0.32732
GDP per capita	64.50	0.00000	151.89	0.30405
Socioeconomic factors				
Schooling	53.40	0.00000	78.22	0.00292
Income	49.10	0.00000	80.14	0.00000

GDP: gross domestic product.

### Effect modifiers of the temperature-mortality association

This heterogeneity could be explained by different local factors that modify the association. We tested three groups of possible effect modifiers (geographic, urban and socioeconomic). Table 6 presents the heterogeneity analysis results.

By including geographic factors, heterogeneity of the temperature-non-accidental mortality association was partially explained mainly by the annual amplitude of mean temperature with a drop in the I^2^ value to 64.7% in the General group and 61.2% in the Older Adult group. Analyses conducted with data from the Older Adult subgroups also pointed to this importance, with lower I^2^ values in the three groups of causes. For all mortality groups tested, the model including the annual amplitude of mean temperature obtained the best model fit among the metapredictive variables considered, the lowest AIC and a significant Wald test. The model tested with the metapredictor variable latitude reached I^2^ values close to those obtained with the amplitude of mean temperature.

Regarding urbanization factors, including the metapredictive variables demographic density and GDP per capita (Table 7) into the model changed very little the I^2^ value. Both models had the highest AIC and nonsignificant Wald tests, except for GDP per capita for the circulatory group (Wald test, p = 0.03).

**Table 7:** Demographic density, gross domestic product (GDP) per capita, schooling and income values of each metropolitan area, Brazil.

Metropolitan area	Population density (inhabitants/km^2^)	GDP per capita (BRL)	Schooling (%) *	Income (%) **
Central-West				
Goiânia	233.66	11,490.39	18.00	61.06
Vale do Rio Cuiabá	39.36	12,324.79	16.45	70.62
Northeast				
Aracaju	1,155.90	10,688.77	17.23	71.62
Campina Grande	101.87	5,823.40	12.48	86.28
Cariri	161.95	5,344.22	16.57	85.36
Feira de Santana	97.58	5,989.28	13.40	84.55
Fortaleza	689.02	10,503.78	18.24	81.79
Grande São Luís	242.94	4,779.35	16.92	83.68
João Pessoa	777.98	12,701.20	13.60	80.74
Maceió	282.46	6,888.31	13.56	80.71
Natal	570.88	8,586.15	15.18	79.03
Palmeira dos Índios	71.60	4,376.71	11.77	87.41
Patos	37.60	4,946.27	12.70	85.47
Recife	1,853.93	17,440.79	16.62	75.20
Salvador	730.31	44,361.67	17.15	70.76
Sudoeste Maranhense	40.59	5,105.70	17.29	81.66
North				
Belém	775.43	7,970.23	20.81	72.91
Capital	11.00	12,056.52	14.23	79.02
Gurupi	4.49	15,891.93	15.86	74.23
Macapá	42.52	10,954.47	15.67	72.47
Manaus	17.87	9,319.04	16.69	79.41
Palmas	13.94	12,442.85	16.02	73.98
Southeast				
Belo Horizonte	464.97	27,143.01	17.26	62.90
Grande Vitória	1,101.57	25,619.64	17.73	54.30
Ribeirão Preto	102.99	20,183.25	18.79	50.74
Rio de Janeiro	2,556.22	14,884.83	19.60	59.77
São Paulo	3,215.82	25,795.12	19.31	51.76
Sorocaba	207.72	25,751.25	18.81	51.99
Vale do Aço	127.61	9,460.22	15.64	74.00
Vale do Paraíba e Litoral Norte	128.03	17,863.77	17.89	58.58
South				
Campo Mourão	26.12	14,891.16	16.61	64.99
Carbonífera	112.31	18,987.96	18.78	48.77
Chapecó	49.46	18,335.12	17.30	49.25
Contestado	34.55	18,111.30	17.16	50.97
Curitiba	348.76	19,179.11	17.59	56.73
Florianópolis	151.90	16,933.64	18.04	47.70
Lages	13.67	16,400.93	16.44	64.57
Londrina	69.58	15,009.61	16.78	56.30
Maringá	103.88	14,322.12	17.75	52.55
Porto Alegre	750.14	28,781.37	20.52	46.05
Serra Gaúcha	110.42	25,485.10	19.54	35.60
Vale do Itajaí	131.08	22,861.73	20.14	33.91

* Percentage of individuals over 10 years old with complete elementary school;

** Percentage of individuals with no income or with an income of up to one minimum wage.

Socioeconomic factors (Table 7) reduced I^2^ values when included in the prediction models of non-accidental mortality groups and Older Adult subgroups. Models that included the income factor found lower I^2^ values when compared with the schooling factor.

**Figure 3:**
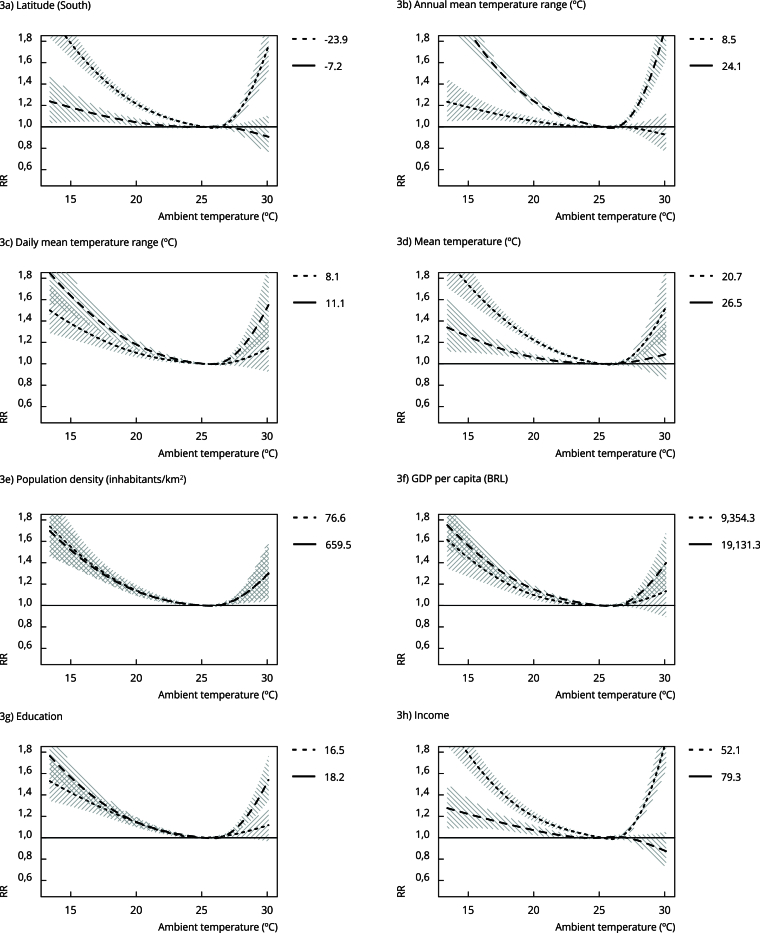
Cumulative 21-day lag temperature-circulatory mortality association curve of the Older Adult subgroups estimated for Brazil according to the 25th and 75th percentiles of each metapredictor.

GDP: gross domestic product; RR: relative risk.

Figure 3 shows the estimated temperature-mortality associations for the 25th and 75th percentiles values of the metapredictors included in each meta-regression model for the Older Adult circulatory mortality subgroup. We note steeper curves and higher relative risks associated with extreme temperatures for: locations at lower latitudes (towards the South), high annual amplitudes of mean temperature, lower average mean temperatures and lower income index value. Despite the proximity between the estimated temperature-mortality association curves for the variables daily amplitude of mean temperature and schooling, the results indicate that higher levels of these predictors lead to higher mortality risks. The other mortality groups studied had curve patterns similar to those of the cardiovascular subgroup, except for the metapredictor GDP per capita which was significant only in this group.

## Discussion

Using the DLNM method ^26^ ^,^ ^37^ allowed us to capture the nonlinear and lagged relation dependent on the temperature-mortality association in Brazil. Our research advances in relation to previous studies ^2^ ^,^ ^23^ ^,^ ^28^ ^,^ ^39^ by presenting the effect modifiers of extreme temperatures on non-accidental mortality in the general population and on four mortality causes among older adults, in addition to using a large number of metropolitan area distributed across the national territory.

Our results show the effects of extreme temperatures on the increased risk of mortality for non-accidental causes in the general population and for non-accidental, circulatory, respiratory and other causes in older adults in the metropolitan area, as well as in the Central-West, Northeast, Southeast and South regions. Effect shape and intensity and MMT/MMP values varied between the locations and causes studied. Geographic aspects, annual amplitude of the mean temperature and latitude were the effect modifier factors of the temperature-mortality association with the greatest impact, followed by income and, more discreetly, schooling. This modulating effect was found for the General and Older Adult groups, as well as for all causes of death subgroups.

The increased relative risk of non-accidental mortality associated with higher and lower temperature extremes found corroborates studies from China ^34^, United States ^14^ and South Africa ^10^, which also included several locations. Such association variability between the analyzed locations has already been shown by studies in Brazil ^2^ and other countries ^8^ ^,^ ^16^ ^,^ ^40^.

Effects of extreme, high and low ambient temperatures on circulatory mortality in older adults were greater in locations southern and southeastern Brazil, as shown by previous studies in Brazil ^28^ ^,^ ^39^ and other locations ^3^. Greatest impact of cold for this group was similar to previous studies with the Brazilian general population ^28^ and in other locations ^10^ ^,^ ^32^ ^,^ ^41^. Hospitalization for cardiovascular disorders also suffers greater influence of cold ^42^. Multiple physiological mechanisms are pointed out as promoters of cardiovascular responses induced by temperature changes, such as the high reactivity of the sympathetic nervous system and the renin-angiotensin system activated by cold, dehydration mediated by heat and cold, as well as systemic inflammatory response induced by heat stroke ^29^ ^,^ ^43^.

Our findings confirmed the effect of extreme ambient temperature on respiratory mortality in older adults, corroborating other studies that also investigated this outcome ^10^ ^,^ ^14^ ^,^ ^32^ ^,^ ^44^. Effects of heat and cold on other causes of death in the Older Adult group confirm the findings of previous studies that considered this group ^10^. This population includes mortality from genitourinary, digestive and endocrine diseases that are sensitive to extreme ambient temperatures ^44^.

The cold and heat effects (RRs) for all causes of mortality had different and increasing values in southern Brazil, with greater impact of extreme ambient temperature in the South and Southeast. These data corroborate previous studies that show response heterogeneity between regions of a given territory ^9^ ^,^ ^15^.

Of the effect modifiers of the temperature-mortality association tested, geographic factors had the greatest impact. Range of ambient temperature, latitude and mean ambient temperature helped to explain the heterogeneity between locations, the first showing the greatest effect. A previous study on cardiovascular mortality in Brazil identified the influence of mean temperature amplitude on the temperature-mortality association ^28^. Significant effect of latitude on the heterogeneity of the associations between ambient temperature and mortality has been previously reported for non-accidental ^11^ ^,^ ^32^ ^,^ ^33^ ^,^ ^34^ and cardiovascular ^3^ mortality. The latitude indicator may represent the effect generated by the annual variation in mean temperature, since the amplitude of mean temperature is greater in the southernmost metropolitan areas.

Variations in the estimated RRs of the temperature-mortality association and the modulating effect of mean temperature amplitude may be related to different physiological adaptation (acclimatization) responses to different climatic situations ^15^.

MMT data from our study point in the same direction. MMT is a characteristic aspect of the temperature-mortality association and how it can be influenced by many factors ^45^. MMT is the temperature with the least effect on the mortality rate ^38^, thus being a threshold and would be related to people’s ability to adapt to the local climate. Here, the estimated MMT values for the General group and the Older Adult group varied between locations, with higher values in places close to the Equator and decreasing along their distance, similar to previous studies ^9^ ^,^ ^32^ ^,^ ^35^ ^,^ ^38^. Locations with smaller ambient temperature ranges are close to the equator, thus its residents are consistently exposed to higher temperatures. Individuals routinely exposed to higher temperatures could develop acclimatization to this condition, with more efficient and less pronounced physiological responses to temperature extremes ^43^.

Additionally, there could be other behavioral adaptations (e.g., use of air conditioning/heater) of the populations to the local climate ^34^ that could explain this heterogeneity.

Among the urban factors, population density had no effect on any of the groups, contrary to previous studies that reported its influence and showed that the effect of heat on mortality was greater in places with higher population density ^8^ ^,^ ^35^. GDP per capita was a modulating factor of the temperature-mortality association only for the circulatory subgroup, but slightly reduced heterogeneity. Higher GDP per capita values had higher RR, in line with previous studies that showed the influence of this effect modifier ^32^. A systematic review on the effect modifiers of the temperature-mortality association reported weak to limited evidence for the influence of community factors like population density, heating system, health facilities, proximity to water, housing quality, and level of air pollution, and limited or suggestive evidence for socioeconomic status, latitude, urban/rural, air conditioning, climatic condition, proportion of green areas or vegetation, and previous winter mortality ^17^.

Socioeconomic factors, schooling and income moderately influenced the temperature-mortality association between locations in all the groups studied. Schooling level was measured by the percentage of individuals with complete primary education, with higher RR values found for places with higher percentage values. Some studies show greater susceptibility of illiterate individuals with respect to the ambient temperature effect on non-accidental mortality ^7^ ^,^ ^32^ ^,^ ^46^, while other studies show that the percentage of individuals with a schooling level lower than the ninth grade (modifying factor) does not explain the heterogeneity between cities ^11^. In this case, the difference could be explained by the analysis approach and difference in schooling stratification. Studies on the cardiovascular temperature-mortality association that included the schooling factor presented divergent results ^3^.

Regarding the income indicator, we note that places where a higher percentage of people have an income level equal to or less than one minimum wage or no income show a greater effect of ambient temperature on mortality. Previous studies that accounted for individuals’ poverty reported its influence only on heat effects ^11^ ^,^ ^34^, while studies focusing on cardiovascular mortality presented divergent results in relation to this indicator ^3^.

Study limitations includes those inherent to using secondary databases. Missing climate data throughout the time series in some metropolitan areas was minimized by using imputations for locations with more than one climate monitoring station. Another limitation was the lack of adjustments due to local air pollution levels given the lack of data in most locations studied. However, previous studies that analyzed the influence of air pollution on the temperature-mortality relation showed slight or no change in effects ^11^ ^,^ ^14^ ^,^ ^46^. Thus, confounding in this case would be low ^46^.

We did not analyze the role of other climatic factors and events ^13^ ^,^ ^47^ such as precipitation, excessive rainfall, drought periods, heat and cold waves, which also occur in Brazil and could contribute to mortality events. Future studies should seek to verify the influences of these factors.

Research on factors modifying the effect of extreme ambient temperature on mortality is important to identify vulnerabilities that could amplify this effect and which can be minimized with appropriate mitigation proposals. Such adaptations, whether undertaken by a person or an institution, could reduce the impact of this climate factor ^48^ in population mortality, especially for those most susceptible such as older adults ^17^.

Our study used a larger number and size of locations in Brazil to address the effect and its modifiers of the temperature-environment association on non-accidental, circulatory, respiratory and other causes mortality in older adults. Besides reinforcing the findings of previous studies, this work enables visualizing places and populations with more immediate needs for climate adaptation actions.

## Data Availability

Daily meteorological indicators (mean, maximum, minimum temperature and relative humidity) and location (latitude) of the meteorological station were extracted from the Meteorological Database for Teaching and Research (BDMEP, acronym in Portuguese; https://bdmep.inmet.gov.br/), Brazilian National Institute of Meteorology (INMET, acronym in Portuguese).
