# Supplementary material for: Effect modifiers of the temperature-mortality association for general and older adults population of Brazil’s metropolitan areas
Source: Cad Saude Publica. 2025 Feb 24;41(2):e00042524. doi: 10.1590/0102-311XEN042524 (PMC11863635; doi:10.1590/0102-311XEN042524)
Supplement: Translation

# Translation: Modificadores de efeito da associação temperatura-mortalidade da população total e idosa das regiões metropolitanas do Brasil

**DOI:** 10.1590/0102-311XPT042524

**Authors:** Cristiane Aschidamini, Antônio Carlos Monteiro Ponce de Leon

**Affiliations:** 1 Universidade do Estado do Amazonas, Manaus, Brasil.; 2 Centro Biomédico, Universidade do Estado do Rio de Janeiro, Rio de Janeiro, Brasil.

**Keywords:** Temperatura Ambiente, Mortalidade, Modificador do Efeito Epidemiológico, Efeitos do Clima

## Abstract

O efeito da temperatura ambiente na mortalidade varia entre locais e populações sugerindo a presença de modificadores de efeito dessa associação. O objetivo deste estudo foi analisar a influência de fatores geográficos, urbanos e socioeconômicos no efeito da temperatura ambiente na mortalidade não acidental da população geral e idosa das regiões metropolitanas brasileiras, e a associada às mortalidades circulatória, respiratória e outras causas dos idosos. Os efeitos dessa associação foram estimados para cada grupo nos 42 locais via modelo aditivo generalizado combinado ao modelo não linear de *lag* distribuído. A seguir foi realizada uma metanálise para estimar os efeitos a nível Brasil e suas regiões. A influência dos modificadores de efeito foi determinada via metarregressão. Os riscos relativos estimados da associação temperatura-mortalidade variaram entre os locais do território brasileiro. Os efeitos do calor na mortalidade não acidental a nível nacional para o grupo Geral e Idoso foram de 1,09 (IC95%: 1,04-1,15) e 1,13 (IC95%: 1,07-1,20), e os do frio foram de 1,26 (IC95%: 1,21-1,32) e 1,30 (IC95%: 1,24-1,36), respectivamente. Observa-se um maior efeito do frio do que do calor em ambos os grupos. Para todas as causas de óbito, os efeitos do calor e do frio foram maiores nas regiões Sudeste e Sul do Brasil. O fator que melhor explicou a heterogeneidade entre os locais foi a amplitude da temperatura média, seguido de latitude, renda e educação. Assim, a implementação de medidas adaptativas para reduzir os efeitos da temperatura ambiente na mortalidade depende do perfil de cada local.

## Introdução

As temperaturas ambientes altas e baixas estão relacionadas a aumentos nos casos de emergências médicas, hospitalização e óbitos ^1^. Essa associação temperatura-mortalidade foi observada para mortalidade geral ^1^ ^,^ ^2^, por doenças cardiovasculares ^3^ ^,^ ^4^, por doenças respiratórias ^5^, e por doenças cerebrovasculares ^4^ ^,^ ^6^, em locais da Europa ^7^, Ásia ^8^ ^,^ ^9^, África ^10^, América do Norte ^11^ e América Latina ^12^, evidenciando assim um problema de saúde global que pode ser amplificado com os eventos de mudança climática ^13^.

Esses riscos de mortalidade relacionados à temperatura ambiente variaram entre locais ^4^ ^,^ ^9^ ^,^ ^11^ ^,^ ^12^ ^,^ ^14^ ^,^ ^15^ ^,^ ^16^, o que pode ser devido à variabilidade de características individuais e comunitárias ^17^, características socioeconômicas ^7^, geográficas ^16^ ou diferentes respostas de adaptação ^2^. Esses fatores modificadores do efeito da associação temperatura-mortalidade foram investigados em estudos anteriores, concentrados em áreas da América do Norte, Ásia e Europa ^17^. Contudo, esses estudos mostraram resultados divergentes e poucos compararam regiões geográficas e climáticas distintas ^17^ ^,^ ^18^.

O Brasil também é vulnerável aos efeitos das temperaturas extremas, com fração de mortalidade atribuída a estas variando de 2,04% a 7,08% entre as capitais brasileiras ^2^. Essas condições de estresse térmico estão associadas a maiores taxas de hospitalização ^19^ ^,^ ^20^ e mortalidade por doenças cardiovasculares e respiratórias nas capitais brasileiras ^21^.

Localizado na América do Sul, o Brasil tem mais de 203 milhões habitantes distribuídos em aproximadamente 8,5 milhões de km^2^ e é um país emergente que ainda apresenta poucos locais em seu território com políticas ativas consistentes de adaptação ou mitigação às mudanças climáticas ^22^. O Brasil tem grande extensão territorial, diversidade socioambiental e sociodemográfica, apresentando-se como um local de estudo adequado para expandir as pesquisas nessa área.

Há poucos estudos em nível nacional abordando o efeito de temperaturas extremas na mortalidade ^23^, principalmente sobre a população idosa brasileira, grupo em expansão e vulnerável frente aos efeitos da temperatura ambiente ^17^ ^,^ ^23^. Além disso, esses estudos não abordaram os efeitos sobre populações residentes em regiões metropolitanas brasileiras. Tal recorte espacial de grande urbanização é constituído por um conjunto de municípios contíguos e socioeconomicamente integrados a uma cidade central, com serviços públicos e infraestrutura comum, e concentram um terço da população do país, 60% do produto interno bruto (PIB) nacional e 70% da pobreza urbana ^24^. Estudos nesses locais permitem a análise de dados de um maior contingente de pessoas vivendo em áreas urbanas, fator também relacionado a maior vulnerabilidade à temperaturas extremas ^18^.

Compreender essa associação e seus fatores moduladores permite o planejamento de intervenções adequadas para subpopulações com diferentes status de vulnerabilidade, além de prover predições confiáveis dos efeitos das mudanças climáticas ^2^ ^,^ ^17^.

Para contribuir com essas questões, este estudo teve como objetivo estimar o efeito da temperatura ambiente na mortalidade não acidental da população em geral e de idosos ao longo do território brasileiro, bem como analisar a influência da diversidade geográfica, urbana e socioeconômica nessa relação.

## Métodos

### Desenho do estudo

Foi realizada uma análise de série temporal de dados diários de mortalidade e condições meteorológicas, referente ao período de 1º de janeiro de 2000 a 31 de dezembro de 2014, de 45 regiões metropolitanas do Brasil, localizadas nas regiões Centro-oeste (2), Nordeste (16), Norte (7), Sudeste (8) e Sul (12). As outras 29 regiões metropolitanas foram excluídas por não apresentarem estação de coleta de dados climáticos.

### Dados de mortalidade

Os dados de mortalidade foram obtidos no Sistema de Informações sobre Mortalidade (SIM) do Departamento de Informática do SUS (DATASUS). A mortalidade diária não acidental (grupo Geral) é representada pela contagem total de óbitos excluindo as causas externas (Classificação Internacional de Doenças, 10ª revisão [CID-10]: código A00-R99). O grupo de idosos (grupo Idoso) foi formado com os dados dos óbitos de indivíduos com 60 anos ou mais.

Buscando verificar a diversidade de efeitos entre as causas de óbito, foi analisada a associação entre mortalidade por temperatura devido a causas circulatórias, respiratórias e outras causas em idosos. Para isso os dados de mortalidade do grupo Idoso foram estratificados por causa do óbito, formando os subgrupos: circulatório (doenças do aparelho circulatório: código I00-I99), respiratório (doenças do aparelho respiratório: código J00-J99) e outras causas (códigos A00-H95 e K00-R99).

A presença de baixos números de óbitos na série temporal podem levar à imprecisão das estimativas ^25^. A presença de dados imprecisos na primeira fase da análise estatística pode gerar viés nos dados estimados na segunda fase ^26^. Para minimizar possíveis erros nas estimativas combinadas subsequentes, aplicaram-se critérios de exclusão. Primeiramente foram excluídas regiões metropolitanas com número de óbitos por dia inferiores a 1,5, permanecendo para as análises dos grupos Geral e Idoso 43 regiões metropolitanas. Finalmente, uma região metropolitana foi excluída devido a inconsistências numéricas (problemas numéricos na estimação). Para os subgrupos do grupo Idoso, com menores contagem de mortes, também foram excluídas as regiões metropolitanas com média diária de óbitos abaixo de 1 em todos os três subgrupos. Assim, para as análises dos subgrupos do grupo Idoso foram consideradas 38 regiões metropolitanas.

### Dados meteorológicos

A temperatura média diária (ºC) foi escolhida como variável de exposição a ser analisada, por representar a exposição durante todo o dia e noite, ter o melhor desempenho na previsão dos efeitos da temperatura sobre a mortalidade ^9^, e porque as diferentes medidas de temperatura têm habilidades preditivas similares ^10^ ^,^ ^27^.

O indicador de umidade relativa média do ar (%) utilizado foi a média do dia atual e do dia anterior. Este foi incluído na análise como fator confundidor, assim como em estudos anteriores ^23^ ^,^ ^28^. A umidade influencia a temperatura por meio da modulação da sensação térmica ^29^ ^,^ ^30^. Ela também influencia o desenvolvimento de doenças respiratórias ^30^ e cardiovasculares por meio do seu efeito sobre o estresse térmico, a desidratação e a proliferação de vetores de doenças ^29^ ^,^ ^30^.

A presença de dados faltantes de temperatura compensada e umidade relativa médias pôde ser minimizada para cinco locais que apresentavam duas ou mais estações metropolitanas. Nestas foi realizado a imputação de valores faltantes usando o pacote *mtsdi* ^31^ da plataforma R (http://www.r-project.org). Foi utilizado um modelo com *spline* cúbica não paramétrica com 8 graus de liberdade (g.l.) para predição dos dados a serem imputados. Ocorreu imputação apenas para os dias em que havia pelo menos uma observação por estação climática e não poderia haver perda de dados em uma sequência superior a três dias.

Estudos anteriores têm reportado a influência das variações da temperatura ^28^ ^,^ ^32^, no dia e no ano, e das localizações, latitude ^11^ ^,^ ^28^ ^,^ ^33^ e região geográfica ^15^ ^,^ ^28^, na associação temperatura-mortalidade. Assim, a média anual da temperatura média, amplitude anual da temperatura média, amplitude diária da temperatura (calculada a diferença entre temperatura máxima e mínima diária), latitude e região geográfica brasileira foram incluídas no modelo como fatores geográficos modificadores da associação temperatura-mortalidade.

### Dados urbanos e socioeconômicos

Características individuais e comunitárias foram identificadas como fatores modificadores da associação temperatura-mortalidade ^17^. Entre os fatores a nível individual temos o nível educacional ^7^ ^,^ ^11^ ^,^ ^32^ e renda ^11^ ^,^ ^18^ ^,^ ^34^. A densidade demográfica ^8^ ^,^ ^18^ ^,^ ^32^ ^,^ ^35^ e PIB *per capita* ^18^ ^,^ ^32^ são fatores comunitários que caracterizam o nível de desenvolvimento urbano.

Assim, os dados socioeconômicos e de desenvolvimento urbano foram incluídos no modelo como fatores modificadores da associação temperatura-mortalidade. Esses dados foram coletados do *Censo Demográfico* de 2010 (https://censo2010.ibge.gov.br/) do Instituto Brasileiro de Geografia e Estatística (IBGE) para cada município, e posteriormente foram calculadas as respectivas médias das regiões metropolitanas.

Como indicadores urbanos utilizou-se densidade demográfica (habitantes/km^2^) e o PIB *per capita* (R$). Como indicadores socioeconômicos utilizou-se renda (percentual de indivíduos sem renda ou com renda de até um salário mínimo) e escolaridade (percentual de indivíduos maiores de 10 anos com Ensino Fundamental completo). Para cada região metropolitana foi estimado a média anual de cada variável numérica e realizada a média do período estudado, sendo este o valor utilizado nas análises.

### Análise dos dados

A associação temperatura-mortalidade foi investigada separadamente para cada grupo de causas de mortalidade com uma análise em duas etapas.

Na primeira etapa foi realizada uma análise de série temporal para cada região metropolitana e grupo usando o modelo aditivo generalizado (GAM, do inglês *generalized additive model*), assumindo um modelo quasi-Poisson ^36^. Foi incluída uma função *cross-basis* do *distributed lag non-linear models* (DLNM) ^37^ para modelar o efeito não linear e defasado da temperatura ambiente sobre a mortalidade. Essa função foi definida por meio de uma *spline* natural cúbica com três nós internos colocados nos percentis 10, 75 e 90 da distribuição da temperatura específica de cada local, e uma *spline* natural cúbica, com nós dispostos no intercepto e três nós internos igualmente espaçados na escala *log* de valores das defasagens. A janela de análise utilizada foi de 21 dias (máximo de defasagem até 21 dias), definida por permitir estimar a relação defasada da temperatura sobre a mortalidade, menor efeito de antecipação de mortes e possibilidade de confrontar nossos resultados com pesquisas anteriores.

Foram incluídas no modelo de regressão duas *splines thin plate*, uma para ajuste da tendência temporal e sazonal, e uma para a umidade relativa do ar. Para tendência temporal e sazonalidade foram testados os ajustes usando *splines* de 2 a 8 g.l. e a escolha foi baseada no critério de informação de Akaike (AIC, do inglês *Akaike information criterion*) e análise dos resíduos. Para o ajuste de umidade relativa do ar foram testados de 3 a 6 g.l., sendo a escolha feita com base no menor AIC. Por fim, inclui-se uma variável indicadora para cada dia da semana.

A partir desse modelo, foi estimado o percentil de mortalidade mínima (PMM) e a respectiva temperatura de mortalidade mínima (TMM) para cada região metropolitana e grupo de causas ^37^. O efeito da temperatura sobre a mortalidade foi estimado em risco relativo (RR), com efeito do frio estimado através de RR de mortalidade entre o 1º percentil e o PMM, e o efeito do calor estimado do RR de mortalidade entre o 99º percentil e o PMM. Também foram extraídos intervalos de confiança (IC95%) desses valores, sendo utilizado nível de confiança de 95%.

Na segunda etapa foi verificado o grau de heterogeneidade entre os locais e estimada a medida sumária da associação temperatura-mortalidade para todo o país, bem como para as regiões geográficas, para cada grupo de causas. Para isso, utilizando o mesmo modelo de regressão descrito anteriormente, fez-se a redução de toda a associação temperatura-mortalidade acumulada no período de *lag*, extraindo os vetores dos coeficientes estimados e a respectiva matriz de (co)variâncias estimadas para cada local e grupo. Esse passo reduz o número de parâmetros considerados na metanálise do segundo estágio preservando a complexidade da dependência estimada ^26^. O PMM escolhido para ser referência das estimativas foi o valor médio dos PMM estimado nas análises individuais da etapa anterior.

O modelo de metanálise multivariada ^26^ foi utilizado para definir uma associação temperatura-mortalidade média das regiões metropolitanas a nível nacional e para cada região brasileira, e o método de estimação usado foi máxima verossimilhança restrita (REML, do inglês *restricted maximum likelihood*). A quantificação de heterogeneidade nas relações exposição-resposta das regiões metropolitanas foi realizada via teste Q de Cochran e estatística I^2^.

Modelos de metarregressão (*univariable mulivariate meta-regression models*) foram aplicados para avaliar a modificação do efeito atribuíveis a características do lugar. As seguintes variáveis metapreditoras foram analisadas: média, amplitude diária e anual da temperatura média, latitude, densidade demográfica, PIB *per capita*, nível educacional e renda. Os modelos de metarregressão, cada um incluindo um único metapreditor, foram especificados e associações exposição-resposta foram estimadas para os valores dos percentis 25 e 75 das variáveis metapreditoras mencionadas acima. Cada um dos modelos foi testado com respeito à heterogeneidade (teste Q e I^2^) e ao ajuste do modelo (AIC). Utilizou-se o teste de Wald para testar a significância da associação multivariada entre os parâmetros de resultado e cada variável preditora.

Análise de sensibilidade foi realizada testando diferentes parâmetros para a função *cross-basis*. Testou-se o uso de duas funções *splines* (*natural cubic spline* [ns] e *quadratic B-spline* [bs]), e duas distribuições para nós internos (10º, 75º e 90º percentis e 25º, 50º e 75º percentis) da distribuição da temperatura. A decisão pelo melhor modelo foi feita por meio do Q-AIC, um AIC modificado para modelos de verossimilhança ^37^. O modelo escolhido foi o que obteve menor valor no somatório dos Q-AIC de todas as regiões metropolitanas.

As análises estatísticas e gráficos foram realizadas na plataforma R, versão 3.5.1, usando os pacotes *dlnm*, *mgcv* e *mvmeta*.

## Resultados

Um total de 6.483.270 óbitos por causas não acidentais ocorreram entre 2000 e 2014 nas 42 regiões metropolitanas brasileiras consideradas, dos quais 4.290.322 foram de indivíduos maiores de 60 anos. A Tabela 1 apresenta sumários dos dados de mortalidade e clima de cada local. As regiões metropolitanas se estendem da latitude 2º82’ Norte (Região Metropolitana da Capital/Roraima) até 30º5’ (Região Metropolitana de Porto Alegre/Rio Grande do Sul), com temperatura ambiente média variando de 14,87ºC (Região Metropolitana de Lages/Santa Catarina) a 28,11ºC (Região Metropolitana da Capital/Roraima).

**Tabela 1:** Sumário de dados de total de óbitos por grupo e dados climáticos nas regiões metropolitanas de 2000 a 2014, Brasil.

Região Metropolitana	Somatório de óbitos					Dados climáticos			
	Geral	Idosos				Temperatura ambiente (ºC)		**Umidade média *lag01* (%)**	
	Não acidental	Não acidental	Circulatório	Respiratório	Outras causas	Média	DP	Média	DP
Centro-oeste									
Goiânia	131.706	82.553	31.584	14.588	36.381	24,56	1,98	60,74	15,29
Vale do Rio Cuiabá	60.040	35.572	13.575	4.980	17.017	26,44	2,75	72,20	11,20
Nordeste									
Aracaju	50.540	30.876	10.826	3.849	16.201	26,49	1,20	76,92	4,78
Campina Grande	47.411	33.459	10.616	2.598	20.245	23,50	1,43	78,58	7,07
Cariri	39.766	27.367	10.228	3.429	13.710	25,98	1,62	70,05	13,28
Feira de Santana	50.030	32.554	11.732	3.251	17.571	24,39	2,04	80,49	8,74
Fortaleza	225.488	147.691	48.628	19.978	79.085	27,02	0,90	77,42	6,04
Grande São Luís	80.099	47.464	18.096	5.036	24.332	26,96	0,94	82,48	5,47
João Pessoa	81.534	54.591	21.347	6.870	26.374	26,97	1,33	76,73	6,11
Maceió	83.503	49.992	19.760	6.749	23.483	25,28	1,36	79,68	5,62
Natal	83.969	56.514	20.619	6.646	29.249	26,51	1,26	80,52	4,75
Palmeira dos Índios	9.378	6.644	1.979	802	3.863	24,72	1,94	75,47	9,58
Patos	15.202	11.159	4.320	1.128	5.711	27,64	1,56	59,86	11,75
Recife	295.368	193.198	78.787	30.639	83.772	25,97	1,34	77,80	6,79
Salvador	227.941	134.597	48.482	18.918	67.197	25,51	1,55	81,80	5,32
Sudoeste Maranhense	20.227	12.141	4.163	1.125	6.853	27,78	1,45	72,63	11,07
Norte									
Belém	141.727	85.631	29.064	14.623	41.944	26,90	0,87	83,41	5,68
Capital	13.217	6.764	2.419	852	3.493	28,11	1,39	73,90	9,63
Gurupi	8.379	5.511	2.481	618	2.412	26,15	1,66	69,01	14,66
Macapá	20.592	10.392	3.268	1.377	5.747	27,44	1,24	79,99	7,48
Manaus	112.740	62.332	16.763	7.445	38.124	27,36	1,41	82,33	6,51
Palmas	15.857	9.557	4.016	1.191	4.350	27,19	1,64	67,87	15,47
Sudeste									
Belo Horizonte	365.476	236.525	86.501	34.075	115.949	21,84	2,41	64,73	11,30
Grande Vitória	107.848	68.127	28.947	8.009	31.171	24,82	2,36	76,30	6,37
Ribeirão Preto	118.609	84.466	31.641	13.009	39.816	22,45	3,01	68,32	12,43
Rio de Janeiro	1.206.529	832.277	295.955	118.357	417.965	25,22	3,11	71,93	7,52
São Paulo	1.492.580	983.815	403.501	155.806	424.508	20,55	3,40	73,56	8,99
Sorocaba	145.450	100.193	33.220	16.073	50.900	21,21	3,33	74,33	8,64
Vale do Aço	46.841	31.130	10.581	4.402	16.147	21,68	2,63	75,90	8,78
Vale do Paraíba e Litoral Norte	171.065	113.572	37.819	17.035	58.718	20,62	3,36	77,67	7,08
Sul									
Campo Mourão	26.374	19.494	9.010	2.982	7.502	20,36	3,91	83,24	9,18
Carbonífera	35.315	24.259	10.142	3.223	10.894	19,81	4,46	83,71	6,90
Chapecó	25.519	18.547	6.708	3.001	8.838	19,43	4,73	72,86	11,88
Contestado	31.901	22.956	7.621	3.422	11.913	16,77	4,40	76,29	11,42
Curitiba	221.955	147.246	57.287	20.975	68.984	17,82	3,85	80,43	8,10
Florianópolis	59.215	40.232	16.477	5.391	18.364	21,19	3,77	79,05	6,89
Lages	28.541	19.439	6.458	2.917	10.064	14,87	4,27	81,49	8,52
Londrina	78.668	56.761	22.559	8.884	25.318	21,89	3,75	74,30	10,88
Maringá	49.727	36.322	14.538	5.273	16.511	22,57	3,73	68,91	13,13
Porto Alegre	364.965	251.881	93.795	38.466	119.620	19,86	4,87	76,84	8,69
Serra Gaúcha	49.351	36.088	13.012	4.901	18.175	17,23	4,81	76,94	10,65
Vale do Itajaí	42.627	30.433	11.752	4.058	14.623	20,87	3,99	85,95	7,02

DP: desvio padrão.

A associação temperatura-mortalidade não acidental acumulada em 21 dias foi estimada para o Brasil para os grupos Geral e Idoso. No grupo Geral, o RR estimado para o efeito do calor foi 1,09 (IC95%: 1,04-1,15) e para o efeito do frio foi 1,26 (IC95%: 1,21-1,32). No grupo Idoso, o RR estimado para o efeito do calor foi 1,13 (IC95%: 1,07-1,20) e para o frio foi 1,30 (IC95%: 1,24-1,36). Observa-se um maior efeito do frio do que para o calor, em ambos os grupos. Esses valores decorrem da combinação do estimado para cada uma das 42 regiões metropolitanas brasileiras.

### Variabilidade da associação temperatura-mortalidade ao longo do território brasileiro

Ao observar os valores estimados para cada uma dessas regiões metropolitanas (Tabela 2) nota-se o aumento significativo do RR associado a temperaturas extremas, tanto altas quanto baixas, na mortalidade não acidental para os grupos Geral e Idoso em vários locais, principalmente nas regiões Sul e Sudeste do Brasil. Maiores RR foram estimados para o efeito do frio na maioria das regiões metropolitanas. Os valores de RR da associação temperatura-mortalidade no grupo Idoso superam os do grupo Geral na maioria dos locais. Observa-se uma variabilidade nos resultados de RR entre as regiões metropolitanas ao longo do território nacional, que pode ser visualizada na Figura 1, utilizando-se como exemplo as estimativas de RR do grupo Idoso.

**Tabela 2:** Estimativas da associação de temperatura extremas altas (99º percentil x percentil de mortalidade mínima [PMM]) e baixas (1º percentil x PMM) na mortalidade não acidental acumulado por período de 21 dias para cada grupo e local.

Região Metropolitana	Grupo Geral				Grupo Idoso			
	TMM	PMM	RR (IC95%) - calor	RR (IC95%) - frio	TMM	PMM	RR (IC95%) - calor	RR (IC95%) - frio
Centro-oeste								
Goiânia	24,50	0,50	1,16 (1,08-1,24)	1,25 (1,14-1,37)	25,90	0,77	1,23 (1,13-1,34)	1,32 (1,19-1,46)
Vale do Rio Cuiabá	33,40	1,00	1,06 (0,76-1,47)	1,53 (0,93-2,54)	29,70	0,92	1,07 (0,85-1,34)	1,38 (1,04-1,84)
Nordeste								
Aracaju	29,90	1,00	1,03 (0,70-1,53)	1,21 (0,75-1,95)	29,90	1,00	1,07 (0,65-1,78)	1,30 (0,70-2,40)
Campina Grande	26,80	1,00	1,14 (0,96-1,34)	1,55 (1,17-2,04)	26,80	1,00	1,12 (0,92-1,36)	1,46 (1,06-2,01)
Cariri	31,20	1,00	1,07 (0,66-1,75)	1,17 (0,60-2,26)	24,30	0,13	1,10 (0,86-1,42)	1,07 (0,89-1,28)
Feira de Santana	26,40	0,82	1,22 (1,07-1,39)	1,29 (1,09-1,53)	25,80	0,71	1,19 (1,02-1,40)	1,30 (1,07-1,58)
Fortaleza	29,60	1,00	1,11 (0,93-1,31)	1,71 (1,31-2,22)	29,60	1,00	1,09 (0,94-1,26)	1,62 (1,27-2,06)
Grande São Luís	23,00	0,00	1,31 (0,71-2,40)	1,20 (0,78-1,84)	28,10	0,90	1,11 (0,94-1,31)	1,14 (0,87-1,48)
João Pessoa	30,30	1,00	1,09 (0,86-1,37)	1,12 (0,84-1,49)	22,20	0,00	1,14 (0,71-1,83)	1,10 (0,80-1,5)
Maceió	27,60	0,97	1,00 (0,92-1,09)	1,31 (1,01-1,68)	28,70	1,00	1,05 (0,74-1,49)	1,58 (0,99-2,53)
Natal	25,50	0,23	1,12 (0,99-1,27)	1,23 (1,04-1,45)	25,90	0,31	1,12 (0,97-1,30)	1,22 (1,00-1,48)
Palmeira dos Índios	27,20	0,90	1,34 (0,90-2,00)	1,64 (1,14-2,34)	27,40	0,92	1,20 (0,76-1,90)	2,01 (1,31-3,1)
Patos	27,40	0,45	1,15 (0,86-1,54)	1,11 (0,87-1,42)	27,30	0,43	1,28 (0,89-1,84)	1,26 (0,92-1,74)
Recife	29,00	1,00	1,01 (0,93-1,11)	1,11 (0,95-1,31)	27,50	0,88	1,04 (0,96-1,13)	1,11 (0,99-1,24)
Salvador	29,10	1,00	1,08 (0,99-1,18)	1,37 (1,19-1,57)	29,10	1,00	1,07 (0,97-1,19)	1,40 (1,19-1,66)
Sudoeste Maranhense	29,20	0,83	1,03 (0,80-1,32)	1,15 (0,76-1,72)	29,40	0,86	1,18 (0,85-1,64)	1,18 (0,69-2,00)
Norte								
Belém	31,40	1,00	1,22 (0,35-4,29)	1,33 (0,35-5,00)	28,20	0,95	1,01 (0,91-1,11)	1,02 (0,87-1,20)
Capital	32,80	1,00	2,01 (0,14-29,03)	1,72 (0,10-30,20)	23,40	0,00	7,21 (1,26-41,16)	4,03 (1,37-11,8)
Gurupi	32,20	1,00	1,38 (0,41-4,64)	1,70 (0,39-7,35)	27,60	0,81	1,16 (0,77-1,76)	1,21 (0,91-1,60)
Macapá	30,70	1,00	1,07 (0,44-2,61)	1,20 (0,41-3,54)	28,10	0,65	1,17 (0,88-1,57)	1,23 (0,88-1,71)
Manaus	29,10	0,88	1,12 (1,01-1,24)	1,10 (0,99-1,23)	27,60	0,57	1,05 (0,92-1,20)	1,12 (0,97-1,28)
Palmas	29,50	0,90	1,29 (0,92-1,82)	1,06 (0,64-1,76)	22,10	0,00	1,79 (0,41-7,85)	1,56 (0,58-4,17)
Sudeste								
Belo Horizonte	22,10	0,50	1,15 (1,09-1,21)	1,14 (1,09-1,20)	24,00	0,80	1,18 (1,11-1,25)	1,26 (1,20-1,33)
Grande Vitória	31,40	1,00	1,04 (0,60-1,81)	1,44 (0,76-2,73)	27,30	0,82	1,01 (0,86-1,20)	1,39 (1,20-1,61)
Ribeirão Preto	22,60	0,44	1,32 (1,20-1,45)	1,39 (1,24-1,56)	23,10	0,50	1,41 (1,26-1,56)	1,47 (1,28-1,68)
Rio de Janeiro	26,60	0,67	1,34 (1,29-1,40)	1,27 (1,23-1,32)	27,00	0,71	1,45 (1,38-1,51)	1,31 (1,26-1,37)
São Paulo	23,50	0,79	1,19 (1,16-1,23)	1,36 (1,32-1,40)	23,50	0,79	1,26 (1,21-1,31)	1,41 (1,35-1,46)
Sorocaba	24,80	0,86	1,30 (1,19-1,42)	1,62 (1,45-1,82)	24,30	0,81	1,39 (1,25-1,55)	1,60 (1,40-1,82)
Vale do Aço	24,90	0,91	1,13 (0,95-1,35)	1,20 (1,01-1,42)	24,10	0,81	1,11 (0,90-1,37)	1,21 (1,00-1,45)
Vale do Paraíba e Litoral Norte	20,20	0,43	1,21 (0,94-1,55)	1,23 (1,02-1,49)	19,70	0,39	1,28 (0,92-1,76)	1,16 (0,92-1,46)
Sul								
Campo Mourão	21,90	0,57	1,73 (1,28-2,33)	1,59 (1,29-1,95)	22,10	0,59	1,66 (1,16-2,37)	1,63 (1,28-2,08)
Carbonífera	17,90	0,33	1,26 (1,07-1,49)	1,69 (1,44-1,99)	16,70	0,25	1,40 (1,13-1,72)	1,74 (1,42-2,12)
Chapecó	24,90	0,92	1,06 (0,83-1,36)	2,05 (1,59-2,63)	28,90	1,00	1,02 (0,72-1,43)	2,05 (1,11-3,81)
Contestado	21,80	0,90	1,13 (0,91-1,40)	1,94 (1,55-2,42)	22,50	0,94	1,03 (0,83-1,27)	2,16 (1,68-2,80)
Curitiba	19,80	0,66	1,09 (1,01-1,16)	1,42 (1,32-1,52)	20,10	0,69	1,08 (0,99-1,17)	1,44 (1,32-1,57)
Florianópolis	25,10	0,83	1,38 (1,23-1,55)	1,30 (1,11-1,53)	25,20	0,84	1,41 (1,22-1,62)	1,49 (1,23-1,81)
Lages	20,20	0,92	1,05 (0,89-1,24)	1,72 (1,37-2,16)	20,30	0,93	1,06 (0,87-1,29)	1,91 (1,45-2,51)
Londrina	23,20	0,57	1,26 (1,13-1,41)	1,51 (1,33-1,70)	23,40	0,59	1,38 (1,20-1,58)	1,55 (1,34-1,80)
Maringá	24,00	0,59	1,26 (1,09-1,45)	1,51 (1,33-1,71)	25,50	0,79	1,46 (1,22-1,74)	1,59 (1,36-1,86)
Porto Alegre	24,70	0,83	1,45 (1,37-1,54)	1,53 (1,42-1,65)	24,80	0,84	1,55 (1,45-1,66)	1,57 (1,44-1,72)
Serra Gaúcha	22,40	0,87	1,23 (1,08-1,41)	1,52 (1,27-1,81)	22,50	0,88	1,24 (1,05-1,46)	1,61 (1,29-2,01)
Vale do Itajaí	18,60	0,28	1,11 (0,91-1,37)	1,30 (1,06-1,59)	25,30	0,87	1,15 (0,97-1,35)	1,29 (0,96-1,72)

IC95%: intervalo de 95% de confiança; RR: risco relativo; TMM: temperatura de mortalidade mínima.

Nota: em negrito os valores de RR significativos.

**Figura 1:** Distribuição geográfica das estimativas da associação temperatura-mortalidade não acidental no grupo Idoso.

RR: risco relativo; TMM: temperatura de mortalidade mínima.

Na Figura 1, nota-se que a TMM também varia entre as regiões metropolitanas. A TMM é a temperatura ótima ^2^, a temperatura mais confortável ou ideal, a partir dela a mortalidade aumenta ^38^. Os valores estimados de TMM variaram de 17,9ºC a 33,4ºC para o grupo Geral (média: 26,1ºC, desvio padrão - DP: 4,1) e de 16,7ºC a 29,9ºC para o grupo Idoso (média: 25,4ºC, DP: 3,1), com maiores valores de TMM encontrados em latitudes próximas a linha do Equador e menores nas metrópoles situadas mais ao sul.

Os efeitos das temperaturas extremas na mortalidade circulatória, respiratória e outras causas do grupo Idoso por regiões metropolitanas estão apresentados nas Tabelas 3, 4 e 5. A Figura 2 apresenta, por região geográfica, o efeito estimado (RR) das altas e baixas temperaturas nas mortalidades dos subgrupos do grupo Idoso. Pode-se notar diferenças nos RR por região geográfica e causa. O efeito do frio e do calor foi significativo na mortalidade dos três subgrupos do grupo Idoso nas regiões Sul e Sudeste. Na Região Centro-oeste só se observa o efeito do frio. A Região Nordeste apresentou apenas RR de mortalidade circulatória e outras causas associado a baixas temperaturas extremas. A Região Norte não apresentou RR significativos.

**Tabela 3:** Estimativas da associação de temperatura extremas altas (99º percentil x percentil de mortalidade mínima [PMM]) e baixas (1º percentil x PMM) na mortalidade por doenças circulatórias acumulado por período de 21 dias para o grupo Idoso por local.

Região Metropolitana	Mortalidade por doenças circulatórias - grupo Idoso			
	TMM	PMM	RR (IC95%) - calor	RR (IC95%) - frio
Centro-oeste				
Goiânia	26,60	0,85	1,16 (1,01-1,34)	1,38 (1,18-1,61)
Vale do Rio Cuiabá	29,30	0,90	1,26 (0,87-1,82)	1,47 (0,95-2,28)
Nordeste				
Aracaju	29,90	1,00	2,16 (0,99-4,71)	2,15 (0,84-5,50)
Campina Grande	26,80	1,00	1,62 (1,16-2,27)	1,79 (1,03-3,13)
Cariri	31,20	1,00	1,96 (0,76-5,07)	2,51 (0,68-9,30)
Feira de Santana	26,10	0,77	1,09 (0,84-1,41)	1,55 (1,11-2,16)
Fortaleza	26,00	0,13	1,04 (0,90-1,21)	1,27 (1,00-1,62)
Grande São Luís	26,00	0,16	1,16 (0,86-1,56)	1,43 (0,88-2,31)
João Pessoa	30,30	1,00	1,66 (1,05-2,63)	1,59 (0,90-2,80)
Maceió	28,70	1,00	1,25 (0,70-2,23)	2,61 (1,22-5,61)
Natal	25,90	0,31	1,04 (0,85-1,27)	1,49 (1,09-2,02)
Patos	27,10	0,39	1,20 (0,77-1,85)	1,32 (0,85-2,04)
Recife	27,80	0,94	1,01 (0,91-1,12)	1,05 (0,88-1,25)
Salvador	29,10	1,00	1,14 (0,96-1,35)	1,43 (1,09-1,87)
Sudoeste Maranhense	29,60	0,89	1,63 (0,98-2,71)	1,05 (0,47-2,35)
Norte				
Belém	26,00	0,15	1,08 (0,88-1,32)	1,14 (0,86-1,50)
Macapá	22,90	0,00	1,84 (0,29-11,52)	1,44 (0,36-5,74)
Manaus	30,20	0,97	1,00 (0,88-1,15)	1,44 (1,05-1,98)
Sudeste				
Belo Horizonte	24,10	0,82	1,18 (1,06-1,32)	1,34 (1,23-1,46)
Grande Vitória	27,70	0,88	1,12 (0,87-1,43)	1,51 (1,19-1,92)
Ribeirão Preto	24,60	0,75	1,15 (1,01-1,31)	1,48 (1,24-1,75)
Rio de Janeiro	28,20	0,81	1,40 (1,29-1,52)	1,46 (1,35-1,57)
São Paulo	24,20	0,85	1,23 (1,15-1,30)	1,66 (1,55-1,77)
Sorocaba	23,50	0,73	1,50 (1,29-1,74)	1,50 (1,27-1,79)
Vale do Aço	24,50	0,86	1,21 (0,85-1,73)	1,42 (1,05-1,92)
Vale do Paraíba e Litoral Norte	28,30	1,00	1,38 (0,70-2,74)	1,69 (0,60-4,80)
Sul				
Campo Mourão	22,00	0,58	1,35 (0,83-2,18)	1,78 (1,32-2,40)
Carbonífera	25,00	0,88	1,24 (0,95-1,63)	1,61 (1,08-2,39)
Chapecó	25,00	0,92	1,10 (0,68-1,78)	2,44 (1,50-3,98)
Contestado	22,80	0,95	1,02 (0,73-1,43)	2,21 (1,43-3,43)
Curitiba	20,20	0,70	1,05 (0,91-1,21)	1,72 (1,50-1,97)
Florianópolis	25,60	0,87	1,21 (0,96-1,54)	2,06 (1,50-2,83)
Lages	20,60	0,95	1,07 (0,78-1,46)	2,65 (1,64-4,27)
Londrina	23,70	0,62	1,26 (1,02-1,56)	1,86 (1,53-2,26)
Maringá	26,30	0,88	1,51 (1,12-2,03)	1,67 (1,29-2,16)
Porto Alegre	24,70	0,83	1,48 (1,33-1,64)	1,86 (1,62-2,13)
Serra Gaúcha	21,50	0,80	1,42 (1,10-1,84)	1,36 (1,00-1,87)
Vale do Itajaí	17,70	0,21	1,57 (1,17-2,12)	1,69 (1,28-2,22)

IC95%: intervalo de 95% de confiança; RR: risco relativo; TMM: temperatura de mortalidade mínima.

Nota: em negrito os valores de RR significativos.

**Tabela 4:** Estimativas da associação de temperatura extremas altas (99º percentil x percentil de mortalidade mínima [PMM]) e baixas (1º percentil x PMM) na mortalidade por doenças respiratórias acumulado por período de 21 dias para o grupo Idoso por local.

Região Metropolitana	Mortalidade por doenças respiratórias - grupo Idoso			
	TMM	PMM	RR (IC95%) - calor	RR (IC95%) - frio
Centro-oeste				
Goiânia	25,10	0,63	1,34 (1,13-1,61)	1,54 (1,21-1,97)
Vale do Rio Cuiabá	33,40	1,00	1,38 (0,49-3,91)	2,72 (0,59-12,50)
Nordeste				
Aracaju	29,90	1,00	4,65 (1,22-17,71)	5,12 (1,02-25,66)
Campina Grande	25,30	0,92	1,17 (0,76-1,80)	1,73 (0,88-3,39)
Cariri	24,30	0,13	1,28 (0,63-2,60)	1,07 (0,69-1,65)
Feira de Santana	27,00	0,91	1,03 (0,64-1,65)	1,61 (0,88-2,95)
Fortaleza	28,70	0,98	1,00 (0,92-1,09)	1,70 (1,15-2,52)
Grande São Luís	23,00	0,00	2,34 (0,19-29,01)	1,41 (0,24-8,20)
João Pessoa	25,20	0,12	1,24 (0,88-1,74)	1,13 (0,69-1,84)
Maceió	28,70	1,00	2,22 (0,82-5,98)	1,88 (0,51-6,93)
Natal	21,80	0,00	2,59 (0,44-15,08)	1,66 (0,51-5,41)
Patos	23,20	0,00	2,17 (0,27-17,77)	1,77 (0,58-5,42)
Recife	26,20	0,52	1,10 (0,91-1,32)	1,29 (1,01-1,64)
Salvador	29,10	1,00	1,14 (0,87-1,49)	2,09 (1,37-3,21)
Sudoeste Maranhense	26,40	0,17	1,25 (0,49-3,17)	3,95 (0,86-18,22)
Norte				
Belém	23,00	0,00	1,23 (0,29-5,23)	1,05 (0,36-3,10)
Macapá	22,90	0,00	3,86 (0,24-62,9)	3,17 (0,4-25,46)
Manaus	21,90	0,00	4,87 (0,73-32,41)	2,78 (0,61-12,7)
Sudeste				
Belo Horizonte	23,30	0,69	1,33 (1,14-1,56)	1,29 (1,14-1,45)
Grande Vitória	27,10	0,80	1,20 (0,73-1,95)	1,62 (1,07-2,44)
Ribeirão Preto	23,10	0,50	1,58 (1,27-1,96)	2,03 (1,57-2,61)
Rio de Janeiro	23,60	0,32	1,73 (1,52-1,97)	1,27 (1,11-1,45)
São Paulo	22,20	0,65	1,49 (1,36-1,63)	1,28 (1,18-1,40)
Sorocaba	18,40	0,20	1,61 (1,20-2,17)	1,42 (1,04-1,93)
Vale do Aço	21,50	0,44	1,18 (0,75-1,86)	1,82 (1,31-2,54)
Vale do Paraíba e Litoral Norte	21,10	0,51	1,50 (0,80-2,79)	1,25 (0,83-1,90)
Sul				
Campo Mourão	21,20	0,50	1,79 (0,75-4,31)	1,99 (1,20-3,29)
Carbonífera	16,10	0,21	2,13 (1,16-3,91)	2,89 (1,74-4,78)
Chapecó	28,90	1,00	1,57 (0,63-3,95)	4,54 (0,87-23,78)
Contestado	21,40	0,87	1,36 (0,67-2,75)	2,38 (1,26-4,50)
Curitiba	3,30	0,00	1,29 (0,70-2,36)	1,25 (0,84-1,87)
Florianópolis	16,70	0,13	1,79 (1,08-2,97)	1,13 (0,76-1,68)
Lages	10,20	0,14	1,24 (0,68-2,27)	3,22 (1,88-5,52)
Londrina	25,00	0,79	1,55 (1,10-2,19)	1,46 (0,99-2,15)
Maringá	23,30	0,51	1,24 (0,80-1,91)	2,39 (1,65-3,45)
Porto Alegre	24,30	0,80	1,90 (1,61-2,23)	1,60 (1,31-1,95)
Serra Gaúcha	29,70	1,00	1,16 (0,33-4,07)	2,52 (0,51-12,34)
Vale do Itajaí	17,50	0,20	1,63 (0,97-2,74)	1,47 (0,91-2,36)

IC95%: intervalo de 95% de confiança; RR: risco relativo; TMM: temperatura de mortalidade mínima.

Nota: em negrito os valores de RR significativos.

**Tabela 5:** Estimativas da associação de temperatura extremas altas (99º percentil x percentil de mortalidade mínima [PMM]) e baixas (1º percentil x PMM) na mortalidade por outras causas acumulado por período de 21 dias para o grupo Idoso por local.

Região Metropolitana	Mortalidade por outra causas - grupo Idoso		
	TMM	PMM	RR (IC95%) - calor	RR (IC95%) - frio
Centro-oeste				
Goiânia	22,70	0,16	1,15 (1,03-1,29)	1,20 (1,01-1,42)
Vale do Rio Cuiabá	31,20	0,99	1,00 (0,93-1,08)	1,34 (0,86-2,08)
Nordeste				
Aracaju	28,00	0,91	1,06 (0,90-1,26)	1,34 (1,06-1,69)
Campina Grande	25,80	0,97	1,01 (0,92-1,10)	1,40 (1,08-1,82)
Cariri	21,20	0,00	1,66 (0,81-3,44)	1,26 (0,77-2,06)
Feira de Santana	18,50	0,00	1,25 (0,71-2,21)	1,33 (0,93-1,90)
Fortaleza	29,60	1,00	1,16 (0,95-1,42)	1,83 (1,32-2,54)
Grande São Luís	23,00	0,00	1,18 (0,39-3,51)	1,17 (0,55-2,51)
João Pessoa	22,20	0,00	1,16 (0,59-2,27)	1,13 (0,73-1,77)
Maceió	27,00	0,90	1,12 (0,86-1,45)	1,13 (0,82-1,56)
Natal	26,10	0,35	1,14 (0,97-1,35)	1,11 (0,86-1,43)
Patos	31,80	1,00	1,46 (0,57-3,75)	1,68 (0,40-6,96)
Recife	27,70	0,92	1,04 (0,95-1,13)	1,18 (1,04-1,34)
Salvador	29,10	1,00	1,01 (0,88-1,17)	1,24 (0,98-1,56)
Sudoeste Maranhense	22,40	0,00	1,46 (0,25-8,42)	1,32 (0,39-4,47)
Norte				
Belém	31,40	1,00	1,47 (0,17-13,08)	1,57 (0,16-15,49)
Macapá	28,80	0,85	1,02 (0,68-1,54)	1,38 (0,88-2,17)
Manaus	28,00	0,68	1,07 (0,90-1,26)	1,10 (0,93-1,31)
Sudeste				
Belo Horizonte	23,90	0,79	1,13 (1,04-1,24)	1,20 (1,12-1,29)
Grande Vitória	31,40	1,00	1,23 (0,46-3,30)	1,59 (0,50-5,10)
Ribeirão Preto	22,80	0,47	1,38 (1,21-1,57)	1,28 (1,07-1,53)
Rio de Janeiro	26,00	0,61	1,41 (1,32-1,51)	1,28 (1,21-1,37)
São Paulo	22,20	0,65	1,23 (1,17-1,29)	1,26 (1,19-1,33)
Sorocaba	24,70	0,86	1,30 (1,15-1,47)	1,65 (1,38-1,98)
Vale do Aço	28,50	1,00	1,23 (0,70-2,17)	1,66 (0,78-3,53)
Vale do Paraíba e Litoral Norte	20,40	0,45	1,36 (0,97-1,91)	1,29 (1,02-1,65)
Sul				
Campo Mourão	23,30	0,75	1,65 (0,95-2,87)	1,96 (1,34-2,88)
Carbonífera	18,40	0,37	1,22 (0,96-1,55)	1,72 (1,31-2,27)
Chapecó	28,90	1,00	1,12 (0,70-1,78)	1,88 (0,82-4,28)
Contestado	26,50	1,00	1,04 (0,54-2,00)	2,12 (0,82-5,44)
Curitiba	22,30	0,88	1,08 (0,96-1,21)	1,38 (1,20-1,59)
Florianópolis	25,20	0,84	1,52 (1,25-1,87)	1,22 (0,92-1,63)
Lages	17,10	0,66	1,04 (0,81-1,34)	1,31 (0,96-1,81)
Londrina	22,90	0,54	1,47 (1,22-1,77)	1,59 (1,32-1,91)
Maringá	24,20	0,62	1,51 (1,19-1,91)	1,37 (1,10-1,72)
Porto Alegre	25,10	0,86	1,46 (1,34-1,59)	1,38 (1,21-1,57)
Serra Gaúcha	22,80	0,90	1,20 (0,99-1,46)	1,81 (1,41-2,33)
Vale do Itajaí	17,70	0,21	1,18 (0,91-1,53)	1,41 (1,06-1,86)

IC95%: intervalo de 95% de confiança; RR: risco relativo; TMM: temperatura de mortalidade mínima.

Nota: em negrito os valores de RR significativos.

**Figura 2:** Estimativas médias de efeito da temperatura ambiente na mortalidade dos subgrupos do grupo Idoso, acumulada em 21 dias, para o Brasil e cada região brasileira.

IC95%: intervalo de 95% de confiança; PMM: percentil de mortalidade mínima; RR: risco relativo. Nota: o quadrado marca o RR estimado e a linha horizontal marca o IC95%. O tamanho do quadrado é o inverso ao da amplitude do IC95%.

Essa variabilidade nas estimativas das associações temperatura-mortalidade entre as regiões metropolitanas foi testada por meio do teste de heterogeneidade I^2^. A associação temperatura-mortalidade não acidental apresentou o valor de I^2^ igual a 81% no grupo Geral e 79% no grupo Idoso. Menores valores de I^2^ foram encontrados nas análises de heterogeneidade para os subgrupos do grupo Idoso. Quando se incluiu no modelo o fator região geográfica os valores de I^2^ reduziram para todos os grupos (Tabela 6).

**Tabela 6:** Resultados do teste Q de Cochran, estatística I^2^, critério de informação de Akaike (AIC) e teste de Wald para modelos de metarregressão para cada grupo.

Metapreditora	I^2^	Teste Q (valor de p)	AIC	Teste deWald (valor de p)
Grupo Geral				
Mortalidade não acidental				
Sem	81,00	0,00000	26,81	0,00000
Região	65,10	0,00000	54,75	0,00000
Fatores geográficos				
Latitude	67,10	0,00000	31,65	0,00000
Amplitude anual da temperatura média	64,70	0,00000	18,39	0,00000
Amplitude diária da temperatura média	77,00	0,00000	49,89	0,01300
Média da temperatura média	75,90	0,00000	36,64	0,00000
Fatores urbanos				
Densidade demográfica	80,50	0,00000	107,77	0,85977
PIB *per capita*	80,70	0,00000	121,77	0,18142
Fatores socioeconômicos				
Educação	73,10	0,00000	42,46	0,00004
Renda	69,10	0,00000	45,44	0,00000
Grupo Idoso				
Mortalidade não acidental				
Sem	79,00	0,00000	56,76	0,00000
Região	60,00	0,00000	79,33	0,00000
Fatores geográficos				
Latitude	62,90	0,00000	58,43	0,00000
Amplitude anual da temperatura média	61,20	0,00000	49,57	0,00000
Amplitude diária da temperatura média	73,10	0,00000	74,49	0,00033
Média da temperatura média	74,00	0,00000	63,13	0,00000
Fatores urbanos				
Densidade demográfica	78,30	0,00000	138,03	0,80990
PIB *per capita*	78,60	0,00000	149,71	0,06470
Fatores socioeconômicos				
Educação	70,20	0,00000	71,91	0,00002
Renda	65,20	0,00000	71,35	0,00000
Mortalidade por doenças circulatórias				
Sem	68,80	0,00000	116,89	0,00000
Região	40,60	0,00000	128,29	0,00000
Fatores geográficos				
Latitude	40,50	0,00000	116,67	0,00000
Amplitude anual da temperatura média	39,60	0,00000	112,29	0,00000
Amplitude diária da temperatura média	60,40	0,00000	135,82	0,00364
Média da temperatura média	54,90	0,00000	122,86	0,00000
Fatores urbanos				
Densidade demográfica	68,10	0,00000	194,42	0,52725
PIB *per capita*	67,10	0,00000	206,29	0,03023
Fatores socioeconômicos				
Educação	58,80	0,00000	123,70	0,00000
Renda	46,80	0,00000	131,34	0,00000
Mortalidade por doenças respiratórias				
Sem	59,10	0,00000	253,10	0,00000
Região	39,60	0,00000	263,74	0,00000
Fatores geográficos				
Latitude	45,30	0,00000	268,79	0,00000
Amplitude anual da temperatura média	42,30	0,00000	262,80	0,00000
Amplitude diária da temperatura média	48,40	0,00000	263,54	0,00001
Média da temperatura média	52,80	0,00000	268,66	0,00086
Fatores urbanos				
Densidade demográfica	57,70	0,00000	326,94	0,54401
PIB *per capita*	59,00	0,00000	340,70	0,12657
Fatores socioeconômicos				
Educação	50,30	0,00000	267,58	0,00349
Renda	45,70	0,00000	272,33	0,00000
Mortalidade por outras causas				
Sem	63,90	0,00000	56,83	0,00000
Região	42,80	0,00000	85,49	0,00000
Fatores geográficos				
Latitude	45,70	0,00000	72,29	0,00000
Amplitude anual da temperatura média	45,40	0,00000	67,81	0,00000
Amplitude diária da temperatura média	56,70	0,00000	76,90	0,00488
Média da temperatura média	59,60	0,00000	74,68	0,00014
Fatores urbanos				
Densidade demográfica	63,50	0,00000	134,38	0,32732
PIB *per capita*	64,50	0,00000	151,89	0,30405
Fatores socioeconômicos				
Educação	53,40	0,00000	78,22	0,00292
Renda	49,10	0,00000	80,14	0,00000

PIB: produto interno bruto.

### Modificadores de efeito da associação temperatura-mortalidade

Essa heterogeneidade poderia ser explicada por diferentes fatores a nível local que modificam a associação. Três grupos de possíveis fatores modificadores de efeito foram testados neste trabalho (geográficos, urbanos e socioeconômicos) e o resultado das análises de heterogeneidade são apresentadas na Tabela 6.

Ao incluir os fatores geográficos, a heterogeneidade da associação temperatura-mortalidade não acidental foi parcialmente explicada principalmente pela amplitude anual da temperatura média com diminuição do valor I^2^ para 64,7% no grupo Geral e 61,2% no grupo Idoso. A importância desse fator também foi identificada nas análises realizadas com dados dos subgrupos do grupo Idoso, com menores valores de I^2^ nos três grupos de causas. Para todos os grupos de mortalidade testados, o modelo que incluía a amplitude anual de temperatura média foi o que obteve o melhor ajuste de modelo, entre as variáveis metapreditoras consideradas, com menor AIC, além de significativo teste de Wald. O modelo testado com a variável metapreditora latitude alcançou valores de I^2^ próximos aos obtidos com a variável amplitude da temperatura média.

Na dimensão urbanização, as variáveis metapreditoras densidade demográfica e PIB *per capita* (Tabela 7) quando incluídas no modelo alteraram pouquíssimo o valor de I^2^. Ambos os modelos apresentaram os maiores AIC e testes de Wald não significativos, com exceção do PIB *per capita* para o subgrupo circulatório (teste de Wald, p = 0,03).

**Tabela 7:** Valores de densidade demográfica, produto interno bruto (PIB) *per capita*, educação e renda de cada região metropolitana, Brasil.

Região Metropolitana	Densidade demográfica (habitantes/km^2^)	**PIB *per capita* (R$)**	Educação (%) *	Renda (%) **
Centro-oeste				
Goiânia	233,66	11.490,39	18,00	61,06
Vale do Rio Cuiabá	39,36	12.324,79	16,45	70,62
Nordeste				
Aracaju	1.155,90	10.688,77	17,23	71,62
Campina Grande	101,87	5.823,40	12,48	86,28
Cariri	161,95	5.344,22	16,57	85,36
Feira de Santana	97,58	5.989,28	13,40	84,55
Fortaleza	689,02	10.503,78	18,24	81,79
Grande São Luís	242,94	4.779,35	16,92	83,68
João Pessoa	777,98	12.701,20	13,60	80,74
Maceió	282,46	6.888,31	13,56	80,71
Natal	570,88	8.586,15	15,18	79,03
Palmeira dos Índios	71,60	4.376,71	11,77	87,41
Patos	37,60	4.946,27	12,70	85,47
Recife	1.853,93	17.440,79	16,62	75,20
Salvador	730,31	44.361,67	17,15	70,76
Sudoeste Maranhense	40,59	5.105,70	17,29	81,66
Norte				
Belém	775,43	7.970,23	20,81	72,91
Capital	11,00	12.056,52	14,23	79,02
Gurupi	4,49	15.891,93	15,86	74,23
Macapá	42,52	10.954,47	15,67	72,47
Manaus	17,87	9.319,04	16,69	79,41
Palmas	13,94	12.442,85	16,02	73,98
Sudeste				
Belo Horizonte	464,97	27.143,01	17,26	62,90
Grande Vitória	1.101,57	25.619,64	17,73	54,30
Ribeirão Preto	102,99	20.183,25	18,79	50,74
Rio de Janeiro	2.556,22	14.884,83	19,60	59,77
São Paulo	3.215,82	25.795,12	19,31	51,76
Sorocaba	207,72	25.751,25	18,81	51,99
Vale do Aço	127,61	9.460,22	15,64	74,00
Vale do Paraíba e Litoral Norte	128,03	17.863,77	17,89	58,58
Sul				
Campo Mourão	26,12	14.891,16	16,61	64,99
Carbonífera	112,31	18.987,96	18,78	48,77
Chapecó	49,46	18.335,12	17,30	49,25
Contestado	34,55	18.111,30	17,16	50,97
Curitiba	348,76	19.179,11	17,59	56,73
Florianópolis	151,90	16.933,64	18,04	47,70
Lages	13,67	16.400,93	16,44	64,57
Londrina	69,58	15.009,61	16,78	56,30
Maringá	103,88	14.322,12	17,75	52,55
Porto Alegre	750,14	28.781,37	20,52	46,05
Serra Gaúcha	110,42	25.485,10	19,54	35,60
Vale do Itajaí	131,08	22.861,73	20,14	33,91

* Percentual de indivíduos maiores de 10 anos com Ensino Fundamental completo;

** Percentual de indivíduos sem renda ou com renda de até um salário mínimo.

Os fatores socioeconômicos (Tabela 7) reduziram o índice de heterogeneidade I^2^ quando incluídos nos modelos de predição dos grupos de mortalidade não acidental e nos subgrupos do grupo Idoso. Os menores valores de I^2^ foram encontrados nos modelos que incluíram o fator renda, do que os do fator educação.

Na Figura 3 pode-se visualizar as associações temperatura-mortalidade estimadas para os valores dos percentis 25º e 75º das metapreditoras incluídas em cada modelo de metarregressão do grupo Idoso, subgrupo mortalidade circulatória. Pode-se visualizar curvas mais acentuadas e maiores riscos relativos associados a temperaturas extremas para: locais nas latitudes mais baixas (em direção ao sul), altas amplitudes anuais da temperatura média, menores médias das temperaturas médias e menor valor do índice renda. Apesar da proximidade entre as curvas de associação temperatura-mortalidade estimados para as variáveis amplitude diária da temperatura média e educação, os resultados apontam que maiores níveis nesses preditores levam a maiores riscos de mortalidade. Os demais grupos de mortalidade estudados tiveram padrões de curvas similares aos apresentados para o grupo cardiovascular, com exceção para as análises da metapreditora PIB *per capita* que foi significativa apenas neste grupo.

**Figura 3:** Curva de associação temperatura-mortalidade circulatória do grupo Idoso estimada para o Brasil, cumulativo de 21 dias defasagem, de acordo com 25º e 75⁰ percentis de cada metapreditor.

PIB: produto interno bruto; RR: risco relativo.

## Discussão

O uso do método DLNM ^26^ ^,^ ^37^ neste trabalho permitiu capturar a relação não linear e defasada dependente da associação temperatura-mortalidade no Brasil. Este estudo avança em relação a estudos prévios ^2^ ^,^ ^23^ ^,^ ^28^ ^,^ ^39^, pois apresenta os modificadores dos efeitos das temperaturas extremas na mortalidade não acidental da população geral e em quatro causas de mortalidade dos idosos brasileiros, além de utilizar um grande número de regiões metropolitanas distribuídas ao longo do território nacional.

Este estudo evidencia os efeitos das temperaturas extremas no aumento do risco de mortalidade para causas de óbito não acidental na população geral e para causas não acidental, circulatória, respiratória e outras nos idosos das regiões metropolitanas, bem como nas regiões Centro-oeste, Nordeste, Sudeste e Sul do Brasil. O formato, a intensidade do efeito e os valores de TMM/PMM variaram entre os locais e grupos de causas estudados. Os fatores geográficos, amplitude anual da temperatura média e latitude, foram os fatores modificadores de efeito da associação temperatura-mortalidade que tiveram maior impacto, seguidos pelo fator renda e de maneira mais discreta, a educação. Esse efeito modulador foi encontrado para os grupos Geral e Idoso, bem como para todos os subgrupos de causa de óbito.

O aumento do RR da mortalidade não acidental associado a extremos de temperatura maior e menor apresentados neste estudo corrobora os estudos da China ^34^, dos Estados Unidos ^14^ e da África do Sul ^10^, que também incluíram vários locais. A variabilidade dessa associação entre as localidades analisadas já foi exibida em estudos no Brasil ^2^ e em outros países ^8^ ^,^ ^16^ ^,^ ^40^.

Efeitos de temperaturas ambientes extremas, altas e baixas sobre a mortalidade circulatória dos idosos foram maiores em locais situados nas regiões Sul e Sudeste do Brasil, como mostrado em estudos anteriores no país ^28^ ^,^ ^39^ e em outros locais ^3^. O maior impacto do frio para esse grupo foi similar a trabalhos anteriores com a população em geral brasileira ^28^ e em outros locais ^10^ ^,^ ^32^ ^,^ ^41^. A hospitalização por distúrbios cardiovasculares também apresenta um maior efeito do frio ^42^. Múltiplos mecanismos fisiológicos são apontados como promotores de respostas cardiovasculares induzidas pelas mudanças de temperatura, tais como a elevada reatividade do sistema nervoso simpático e sistema renina-angiotensina ativado com o frio, desidratação mediada pelo calor e frio, bem como resposta inflamatória sistêmica induzida por insolação ^29^ ^,^ ^43^.

Neste estudo pode-se confirmar o efeito da temperatura ambiente extrema na mortalidade respiratória dos idosos, corroborando outros estudos que também investigaram esse desfecho ^10^ ^,^ ^14^ ^,^ ^32^ ^,^ ^44^. Os efeitos do calor e do frio na mortalidade por outras causas do grupo Idoso confirmam os achados de estudos anteriores que o consideraram ^10^. Nesse grupo incluem-se mortalidade por doenças geniturinárias, digestivas e endócrinas que são sensíveis à temperatura ambiente extrema ^44^.

Para todas as causas de mortalidade, evidenciou-se efeito do frio e calor com RR estimados de valores diferentes e crescentes em sentido da Região Sul do Brasil, com maior impacto da temperatura ambiente extrema nas regiões Sul e Sudeste. Esses dados corroboram com estudos anteriores que exibem heterogeneidade de respostas entre regiões de seu território ^9^ ^,^ ^15^.

Entre os modificadores de efeito da associação temperatura-mortalidade testados, os fatores geográficos tiveram maior impacto. A amplitude de temperatura ambiente, latitude e média da temperatura ambiente auxiliaram a explicar a heterogeneidade entre os locais, sendo a primeira a com maior efeito. A influência da amplitude da temperatura média na associação temperatura-mortalidade foi identificada em estudo anterior de mortalidade cardiovascular no Brasil ^28^. O efeito significativo da latitude na heterogeneidade das relações entre temperatura ambiente e mortalidade foi relatado anteriormente para mortalidade não acidental ^11^ ^,^ ^32^ ^,^ ^33^ ^,^ ^34^ e cardiovascular ^3^. O indicador latitude pode estar representando o efeito gerado pela variação anual temperatura média, visto que nas regiões metropolitanas mais ao sul a amplitude da temperatura média é maior.

Essas variações dos RR estimados da associação temperatura-mortalidade e o efeito modulador da amplitude da temperatura média podem estar relacionadas a diferentes respostas de adaptações fisiológicas (aclimatação) frente a situações climáticas distintas ^15^.

Os dados de TMM deste trabalho apontam esse mesmo sentido. A TMM é parte característica da associação temperatura-mortalidade e como esta pode ser influenciada por muitos fatores ^45^. A TMM é a temperatura com menor efeito sobre a taxa de mortalidade ^38^, sendo assim um limiar e estaria relacionado a capacidade de adaptação dos indivíduos ao clima local. Neste estudo os valores de TMM estimados para os grupos Geral e Idoso variaram entre os locais, com valores mais altos nos próximos à linha do Equador e reduzindo ao longo do seu distanciamento, similar ao exibido por estudos anteriores ^9^ ^,^ ^32^ ^,^ ^34^ ^,^ ^38^. Os locais com menores amplitudes de temperatura ambiente ficam próximas ao Equador, portanto os residentes desses locais são expostos, consistentemente, a temperaturas mais altas. E indivíduos expostos rotineiramente a temperaturas mais elevadas poderiam desenvolver aclimatação a essa condição, com respostas fisiológicas mais eficientes e menos pronunciadas frente a extremos de temperatura ^43^.

Além dessa aclimatação fisiológica, poderia haver outras adaptações comportamentais (p.ex.: uso do ar-condicionado/aquecedor) das populações frente ao clima local ^34^ que poderiam explicar essa heterogeneidade.

Dos fatores urbanos, a densidade demográfica não teve efeito para nenhum dos grupos, não corroborando os estudos anteriores que relataram influência desse fator e que exibiram que o efeito do calor sobre a mortalidade era maior em locais onde a densidade populacional é maior ^8^ ^,^ ^35^. O PIB *per capita* foi um fator modulador da associação temperatura-mortalidade apenas para o subgrupo circulatório, mas reduziu levemente a heterogeneidade. Maiores valores de PIB *per capita* tiveram maiores RR, indo de encontro a estudos anteriores que mostraram a influência desse modificador de efeito ^32^. Em revisão sistemática que abordou os modificadores de efeito da associação temperatura-mortalidade, houve relato de fracas a limitadas evidências da influência de fatores comunitários, como a densidade populacional, sistema de aquecimento, instalações de saúde, proximidade de água, qualidade da habitação e nível de poluição do ar, e evidências limitadas ou sugestivas para os fatores *status* socioeconômico, latitude, urbano/rural, ar-condicionado, condição climática, proporção de áreas verdes ou vegetação, e mortalidade anterior no inverno ^17^.

Os fatores socioeconômicos, educação e renda influenciaram moderadamente a associação temperatura-mortalidade presente entre os locais em todos os grupos estudados. O nível de educação foi medido pela porcentagem de indivíduos com nível fundamental completo, sendo maiores valores de RR encontrados para os locais onde havia maior valores destes. Alguns trabalhos exibem maior susceptibilidade de indivíduos com nenhum nível educacional ao efeito da temperatura ambiente para mortalidade não acidental ^7^ ^,^ ^32^ ^,^ ^46^, enquanto outros estudos mostram que a proporção de indivíduos com nível educacional menor que a 9ª Série (fator modificador) não explica a heterogeneidade entre as cidades ^11^. Nesse caso, a diferença poderia ser explicada pela abordagem de análise e diferença de estratificação dos níveis educacionais. Os estudos de associação temperatura-mortalidade cardiovascular que incluíram o fator educação apresentaram resultados divergentes ^3^.

No indicador de renda, podemos observar que locais onde há maior porcentagem de pessoas com nível de renda igual ou inferior a um salário mínimo ou sem renda apresentaram maior efeito da temperatura ambiente na mortalidade. Trabalhos prévios que utilizaram a pobreza dos indivíduos relataram sua influência apenas para os efeitos do calor ^11^ ^,^ ^34^, enquanto trabalhos com foco na mortalidade cardiovascular apresentaram resultados divergentes com relação a esse indicador ^3^.

Este estudo apresenta algumas limitações, como as inerentes ao uso de banco de dados secundários. O problema de dados climáticos faltantes ao longo da série temporal em algumas regiões metropolitanas pôde ser minimizado com o uso de imputações para as localidades onde havia mais de uma estação de monitoramento climático. Outra limitação foi a não realização de ajustes devidos aos níveis locais de poluição do ar, decorrente da falta de dados na grande maioria dos locais estudados. Porém, estudos anteriores que analisaram a influência da poluição do ar na relação temperatura-mortalidade mostraram leve ou nenhuma modificação dos efeitos na presença destes ^11^ ^,^ ^14^ ^,^ ^46^. Assim, o confundimento nesse caso seria pequeno ^46^.

Neste trabalho não foi analisado o papel de outros fatores e eventos climáticos ^13^ ^,^ ^47^, como precipitação, excessos de chuvas, períodos de secas, ondas de calor e frio, que também ocorrem no Brasil e que poderiam estar envolvidos nos eventos de mortalidade. Futuros estudos poderiam ser realizados buscando verificar as influências desses fatores.

Pesquisas com fatores modificadores de efeito da temperatura ambiente extrema na mortalidade são importantes para identificarmos vulnerabilidades que ampliariam esse efeito e que podem ser minimizadas com propostas apropriadas de mitigação. Essas adaptações, sejam elas empreendidas por pessoa ou instituição, poderiam reduzir o impacto desse fator climático ^48^ na mortalidade da população brasileira, principalmente para as populações mais susceptíveis como a dos idosos ^17^.

Este trabalho utilizou um maior número e tamanho de locais no Brasil para abordar o efeito e seus modificadores da associação temperatura-ambiente nas mortalidade não acidental, circulatória, respiratória e outras causas da população idosa. Além de reforçar os achados de trabalhos anteriores, este trabalho permite visualizar locais e populações com necessidades mais imediatas de direcionamento de ações de adaptação climática.

## Data Availability

Indicadores meteorológicos diários (temperatura média, máxima, mínima e umidade relativa do ar) e localização (latitude) da estação meteorológica foram extraídos do Banco de Dados Meteorológicos para Ensino e Pesquisa (BDMEP; https://bdmep.inmet.gov.br/) do Instituto Nacional de Meteorologia (INMET).
